# Internal modifications in the CENP-A nucleosome modulate centromeric dynamics

**DOI:** 10.1186/s13072-017-0124-6

**Published:** 2017-04-04

**Authors:** Minh Bui, Mary Pitman, Arthur Nuccio, Serene Roque, Paul Gregory Donlin-Asp, Aleksandra Nita-Lazar, Garegin A. Papoian, Yamini Dalal

**Affiliations:** 1 Chromatin Structure and Epigenetic Mechanisms Unit, Laboratory of Receptor Biology and Gene Expression, CCR, NCI, NIH, Bethesda, MD 20892 USA; 2 Department of Biophysics, University of Maryland, College Park, MD USA; 3 Department of Cell Biology, Emory University, Atlanta, GA USA; 4 Cellular Networks Proteomics Unit, Laboratory of Systems Biology, NIAID, NIH, Bethesda, MD 20892 USA

## Abstract

**Background:**

Posttranslational modifications of core histones are correlated with changes in transcriptional status, chromatin fiber folding, and nucleosome dynamics. However, within the centromere-specific histone H3 variant CENP-A, few modifications have been reported, and their functions remain largely unexplored. In this multidisciplinary report, we utilize in silico computational and in vivo approaches to dissect lysine 124 of human CENP-A, which was previously reported to be acetylated in advance of replication.

**Results:**

Computational modeling demonstrates that acetylation of K124 causes tightening of the histone core and hinders accessibility to its C-terminus, which in turn diminishes CENP-C binding. Additionally, CENP-A K124ac/H4 K79ac containing nucleosomes are prone to DNA sliding. In vivo experiments using a CENP-A acetyl or unacetylatable mimic (K124Q and K124A, respectively) reveal alterations in CENP-C levels and a modest increase in mitotic errors. Furthermore, mutation of K124 results in alterations in centromeric replication timing. Purification of native CENP-A proteins followed by mass spectrometry analysis reveals that while CENP-A K124 is acetylated at G1/S, it switches to monomethylation during early S and mid-S phases. Finally, we provide evidence implicating the histone acetyltransferase (HAT) p300 in this cycle.

**Conclusions:**

Taken together, our data suggest that cyclical modifications within the CENP-A nucleosome contribute to the binding of key kinetochore proteins, the integrity of mitosis, and centromeric replication. These data support the paradigm that modifications in histone variants can influence key biological processes.

**Electronic supplementary material:**

The online version of this article (doi:10.1186/s13072-017-0124-6) contains supplementary material, which is available to authorized users.

## Background

Posttranslational modifications in histones play an important role in chromosome biology. The majority of such modifications discovered exist on the N-terminal tails of histones H3, H2A, H3.3, and H4 [1–3]. N-terminal histone modifications may increase nucleosome turnover [4], be inherited at specific loci [5], alter the binding efficiency of various transcriptionally active or repressive factors [6], and disrupt replication timing [7]. Thus, modifications can potentially influence the fate of the underlying locus. A new area of research has also uncovered covalent modifications within histone fold domains, such as H3K56ac and H3K122ac [8–10]. H3K122 is acetylated at the nucleosome dyad, wherein it alters DNA–histone binding and increases thermal repositioning of the nucleosome in vitro [9]. Concurrently, in vivo experiments demonstrate that H3K122ac promotes nucleosome turnover, thereby stimulating transcription [11, 12]. When mutated, a single residue in the hominid-specific histone variant H3.5, leucine 103, disrupts nucleosome instability both in vitro and in vivo [13]. Further, a single change in the nucleosome, methylation at H3K9, alters replication timing [14]. Thus, internal histone core modifications can alter the nucleosome structure in a manner distinct from that reported for histone tail modifications [6, 15, 16]. Therefore, investigating how such modifications in key histone variants, such as the centromere-specific H3 histone variant CENP-A, can contribute to function is an exciting area of research. Indeed, it has been previously proposed that specific posttranslational modifications could distinguish newly incorporated from old CENP-A and that new CENP-A not appropriately modified could be evicted during late G1 phase [17]. Interestingly, previous work also shows that inhibiting HDACs suppresses the loss of CENP-A at centromeres [18], suggesting that the acetylation of CENP-A plays a role in CENP-A retention.

A plethora of CENP-A modifications have been discovered in recent years [19]. Of these, only three have been reported within the histone fold domain [20–23]. Using epitope-tagged CENP-A, studies have identified phosphorylation of S68 within loop 1 [23], and ubiquitination of K124 near the pseudo-dyad of the nucleosome [21, 22]. We previously analyzed chromatin-bound native CENP-A (nCENP-A) and identified acetylation of K124 (K124ac), which was enriched at G1/S phase [20]. K124 in CENP-A is analogous in location to residue K122 in histone H3, which, as discussed above, has a significant impact on nucleosome structure and function. We previously reported that in advance of replication, CENP-A K124ac and H4K79ac correlate with a transitionary opening of centromeric chromatin fiber [20]. CENP-A K124ac is proximal to the pseudo-dyad DNA of the CENP-A nucleosome and to the CENP-A C-terminus. The latter is required to bind the inner kinetochore protein CENP-C [24]. Therefore, we hypothesized that potential functions of CENP-A K124ac/H4K79ac might be to alter the stability of the CENP-A nucleosome, or, to alter the binding of the kinetochore protein CENP-C. Cumulatively, we suggested such events might be necessary to increase access to centromeric chromatin at the appropriate time in replication. Post-replication, inheritance of specific chromatin states involves a coordinated series of events that include eviction of nucleosomes, followed by rapid reassembly after the passage of the replication machinery [25]. Consequently, constitutive gain or loss of pre-replicative modifications in CENP-A might be expected to influence centromere replication dynamics.

In this report, we dissect the role of CENP-A K124ac in silico and in vivo. First, using all-atom computational modeling, we simulate the presence of K124ac and H4K79ac in the octameric CENP-A nucleosome. We find that these modifications result in a loosening of DNA at the pseudo-dyad, followed by asymmetric site exposure of the terminal ends of the DNA. These DNA dynamics are driven, in part, by an unexpected compaction of the CENP-A protein, accompanied by a locking of the CENP-A C-terminus. Consistent with this finding, further computational analysis of this acetylated CENP-A nucleosome shows dramatically reduced contacts with the key kinetochore protein CENP-C. To examine the function of CENP-A K124ac in vivo, we generated mutants of CENP-A K124, which mimic either the constitutively acetylated (K124Q), or the unacetylated state (K124A) and observe three aspects of centromere dynamics are impacted. First, gain or loss of CENP-A K124ac results in a quantifiable decrease in association of modified CENP-A nucleosomes with native CENP-C. Second, there is a modest accumulation of downstream mitotic errors. Third, relative to wild-type CENP-A, in cells expressing the unacetylatable CENP-A K124A, centromeric foci are delayed in replication timing, whereas the acetyl-mimic K124Q loses mid–late S phase replication bias. Using gel-coupled mass spectrometry analysis of native CENP-A at distinct points spanning G1/S to late S phase, we uncover a switch in modifications of chromatin-bound native CENP-A, from acetylation of K124 at G1/S to monomethylation at S phase. Finally, using two custom antibodies against CENP-A K124ac, our data suggest p300 as a putative HAT, which promotes acetylation of chromatin-bound native CENP-A.

Together, these results suggest a working model wherein cyclical changes in CENP-A K124 modifications facilitate accurate timing of centromeric replication and contribute to mitotic integrity.

## Results

In recent work, using all-atom molecular dynamics (MD), we showed that the 4-helix bundle in the CENP-A nucleosome core particle (CENP-A NCP) is highly dynamic [26], such that CENP-A dimers were found to move antiparallel to each other in a “shearing” motion unique to octameric CENP-A nucleosomes. Earlier experimental work from our laboratory indicated that G1/S CENP-A nucleosomes are enriched in CENP-A K124ac and H4K79ac, and correlated with a transitionary state of the chromatin fiber [20]. To investigate potential dynamics induced by these modifications, we computationally modeled CENP-A K124ac and H4K79ac in the context of the octameric CENP-A nucleosome. Four systems were simulated using all-atom MD: (1) the CENP-A NCP, (2) the acetyl CENP-A NCP (CENP-A K124ac and H4K79ac), (3) the CENP-A NCP bound to the kinetochore protein CENP-C, and (4) the acetyl CENP-A NCP bound to CENP-C. The dynamics and structural time averages of systems 1–4 were compared to determine changes either in proximity of the acetylation sites or even globally. We tested whether these four small charge neutralizations result in detectable perturbations of dynamics in a large system.

### The acetylated CENP-A nucleosome displays a tightening of the histone core

**Fig. 1:**
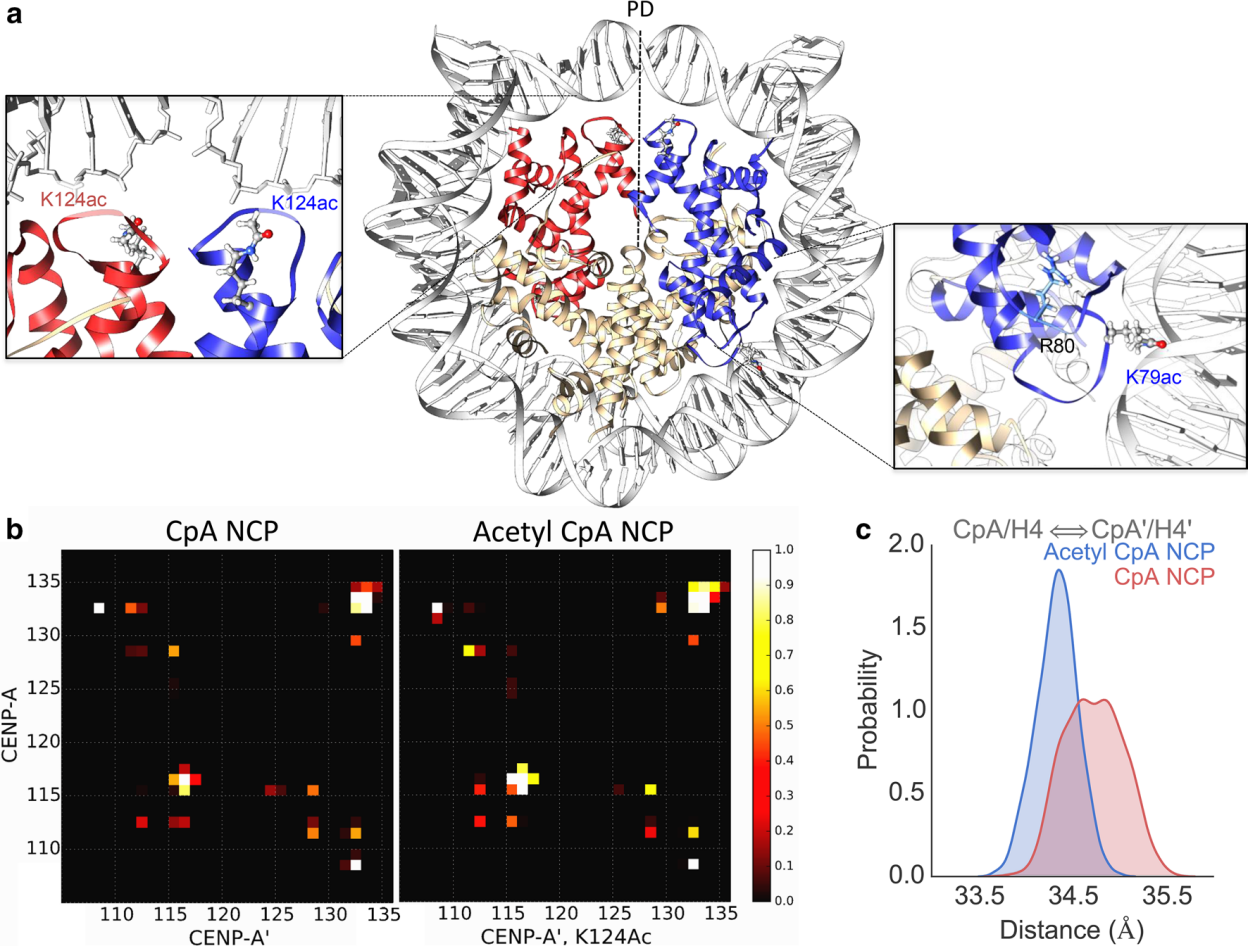
CENP-A nucleosome displays a tightening of the histone core upon lysine acetylation. **a** The starting structure of the acetyl CENP-A NCP is shown with CENP-A and H4 in *red*, CENP-A′ and H4′ in *blue*, and H2A/H2B monomers in *tan*. The pseudo-dyad is shown by a *dotted black line* and marked as *PD*. Overlay’s shown K124ac, K79ac, and CENP-A R80 and the L1 loop in more detail. This structure is after 1-μs simulation of CENP-A, and the production of the starting structure is described (“Methods” section). **b** Contact analysis showing CENP-A (CpA) to CENP-A′(CpA′) interface at the 4-helix bundle. The contact cutoff between Cα atoms was set to 8 Å. An increase in contacts is shown upon acetylation—a decrease in the 4-helix bundle interface distance. A value of 1, *white*, shows a contact formed over all simulation time steps and 0, *black*, never. In the acetyl NCP residues H115, A116, and G117 more frequently form contacts located in the hinge region of the 4-helix bundle. The C-terminus, specifically CENP-A′ residues I132, R133, and G134, forms increased contacts with CENP-A G134. **c** The center of mass (COM) of dimers was measured over all time steps, and the resulting distributions are shown for CENP-A/H4 to CENP-A′/H4′, the acetylated histones. The acetylated system, shown in *blue*, shows a decrease in variance and distance between the two dimer COMs

Due to the loss of positive charge on CENP-A K124ac and H4K79ac at the interface of DNA and histones (Fig. 1a), we hypothesized that DNA contacts with the histone core, and inter-histone repulsion, would both decrease. We tested this hypothesis with detailed contact analysis. Indeed, we found that the acetyl NCP 4-helix bundle interface makes a greater proportion of contacts throughout the simulation (Fig. 1a, b). In the acetyl NCP, residues H115, A116, and G117 frequently form more contacts located in the hinge region of the 4-helix bundle. The constraint on this flexible hinge stabilizes the CENP-A to CENP-A′ interface. As a result, the C-terminus—specifically CENP-A′ I132, R133, and G134—forms more contacts with CENP-A G134 (Fig. 1b).

We next wished to assess whether only the histone interfaces tighten in the acetyl CENP-A NCP or whether the globular histone domains also contract. To investigate this, we calculated the distance between the center of mass (COM) of dimers. In results above, CENP-A/H4 and CENP-A′/H4′ dimers are closer together in the acetyl NCP (Fig. 1c). In other dimer combinations, such as CENP-A/H4 to H2A/H2B, the variance in the distance between dimers decreased, consistent with the rigidification of the histone core upon acetylation. Overall, the observed changes in distances between various dimers show that the histone core is stabilized and tightened in the acetyl NCP.

**Fig. 2:**
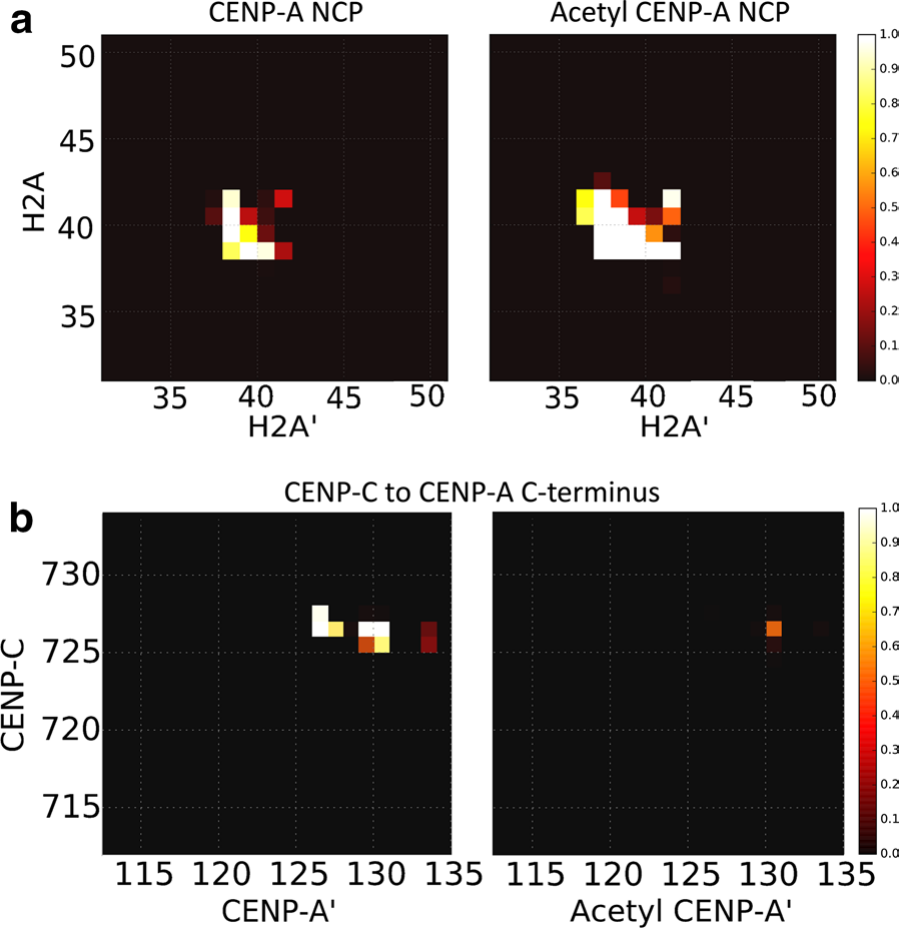
Global compaction of the histone core disrupts CENP-C binding. **a** Contact analysis of the H2A to H2A′ interface on the opposite face of the nucleosome from the acetylations studied. The contact cutoff between Cα atoms was set to 8 Å. A value of 1, *white*, shows a contact formed over all simulation time steps and 0, *black*, never. An increase in contacts is shown upon acetylation consistent with a global compaction of the histone core transduced away from the points of acetylation. **b** To study the effects of the histone core compaction with acetylation, the CENP-C fragment [27] was docked to the nucleosomes and contacts analyzed. The CENP-C fragment is shown to form stable contacts with the CENP-A C-terminal end that are lost upon acetylation

To explore these dynamics in greater detail, we next performed principle component analysis of histone core proteins (PCA^core^) where high-frequency vibrational motions are filtered out within the first few PC modes, thereby revealing large global distortions. In this analysis, the most significant mode of motion, PC1core, revealed a surprising “freezing” or lack of motion at histone interfaces at the pseudo-dyad, adjacent to the K124ac modifications (Fig. 1c; Additional file 1: Movie S1). Because of the decreased histone rocking in the acetyl NCP, even histone interfaces far away from the studied modifications compact, hence, the effect of acetylated lysines transduce to the other face of the nucleosome (Fig. 2a; Additional file 1: Movie S1).

**Fig. 3:**
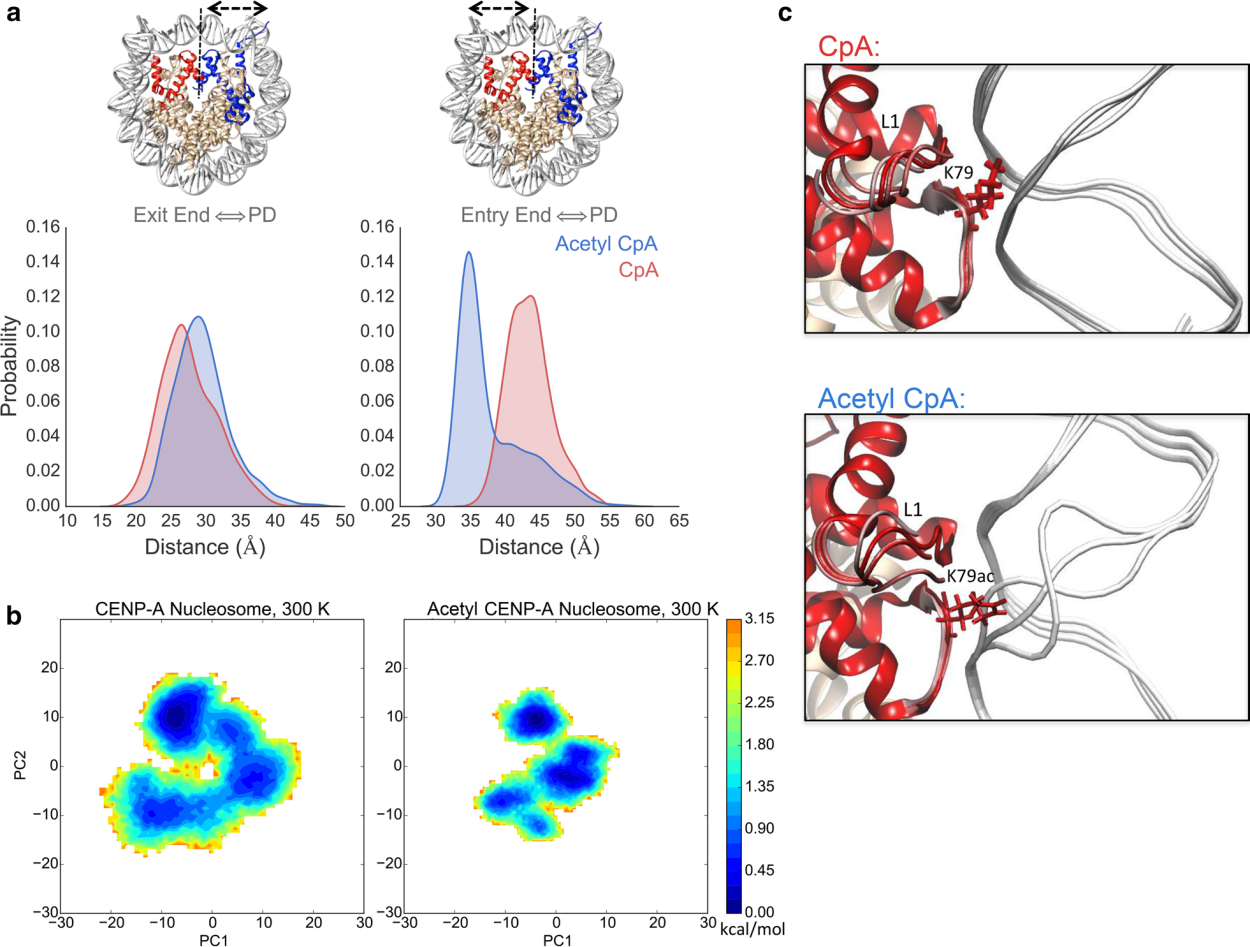
Lysine acetylations asymmetrically loosen DNA entry and exit ends and alter DNA dynamics. **a** The distance between DNA ends to the pseudo-dyad was measured for all time steps and distributions shown. In the structures showing which graph corresponds to which DNA end, the CENP-A NCP is shown with CENP-A and H4 in *red*, CENP-A′ and H4′ in *blue*, and H2A/H2B monomers in tan. The entry end of DNA unwraps more in the acetyl NCP in *blue*. **b** Coarse-grained free energy landscapes are shown for both systems through the projection of PC1^NUC^ and PC2^NUC^ of the whole nucleosomal principal component analysis (PCA^NUC^). Here it is shown that the acetyl NCP landscape becomes more rugged and frustrated. **c** From PC1^NUC^ trajectories, three representative snap shots are shown to illustrate the intra-helical bubble formed in DNA near K79ac that does not occur in unmodified CENP-A. DNA is shown in *white*, and the L1 loop of CENP-A is marked

To compare local structural flexibility, we calculated the root-mean-square fluctuation (RMSF) of Cα atoms (Additional file 2: Fig. S1). Compared to the control, the RMSF of the acetyl CENP-A NCP was locally suppressed in specific regions, namely the C-terminus of CENP-A and the acidic patch of H2A. Both of these regions are targets for CENP-C binding [27]. A diminution in the accessibility of the acidic patch may interfere with H2A D89 binding with CENP-C R717 and R719 [28]. This unexpected and distinct reduction in the availability of the C-terminus of CENP-A and H2A acidic patch emphasizes local changes that result from these acetylations. In order to test whether acetylation modulates CENP-C docking, the CENP-C fragment from the recently solved co-crystal of H3:CENP-A plus CENP-C [27] was docked to each system and simulated for an additional microsecond. As can be seen, the CENP-C fragment forms stable contacts with the C-terminus of CENP-A in the unmodified state, but these contacts are virtually lost upon acetylation (Fig. 2b). The acetylations modeled result in global structural changes that are sufficient to diminish the accessibility of this critical CENP-C docking interface. Interestingly, in the acetyl NCP, the heterotetramer half showing the greatest suppression of RMSF is also the region with increased DNA unwrapping (Fig. 3a, Additional file 2: Fig S1). In summary, our molecular dynamics simulations demonstrate that these CENP-A and H4 acetylations lead to a compaction of the histone core, resulting in a relative burial of the CENP-A C-terminus that is normally targeted by CENP-C. As a consequence, CENP-C binding is greatly diminished (Fig. 2b). To further study the interaction of histones and DNA, we then extended our analysis to the whole nucleosome.

### Specific lysine acetylations increase accessibility of DNA in acetyl NCP

In experiments, we observed that the DNA in both systems, CENP-A and CENP-A K124ac, unwraps asymmetrically, but has enhanced unwrapping in the acetyl NCP (Fig. 3a, Additional file 2: S1). We studied the change in DNA dynamics further with principle component analysis of the whole nucleosome (PCA^NUC^). Visual analysis of the most significant mode, PC1^NUC^, demonstrated a pronounced untwisting motion of DNA ends in the acetyl NCP (Additional file 3: Movie S2). The rigidification of the histone core in the acetyl NCP stabilizes infrequently sampled states of DNA unwrapping in CENP-A (Fig. 3a). We interpret these data to mean that the acetyl NCP system is less freely sampling a larger conformational space, as seen through the attenuation of histone rocking (Additional file 1: Movie S1) and results in a more rugged free energy landscape (Fig. 3b).

Another feature exclusive to PC1^NUC^ of the acetyl NCP is a modulation in the widths of the major and minor grooves of DNA (Additional file 3: Movie S2). The cause for this modulation was a pronounced scissoring motion between helices α2 and α3 of the 4-helix bundle in acetyl CENP-A. We observed a high coherence between the scissoring of the 4-helix bundle with the modulation of the size of the DNA minor grooves (correlation coefficient of 0.82, methods). This suggests that the altered motion of the 4-helix bundle in acetyl CENP-A could promote DNA sliding. PCA^NUC^ also revealed that adjacent to both H4 and H4′ K79ac, two intermittent DNA bubbles formed within the double helix (Fig. 3c; Additional file 3: Movie S2), indicating that these regions of DNA become more susceptible to opening in the presence of H4K79ac. Therefore, the acetylation of H4K79 and CENP-A K124 is sufficient to alter DNA dynamics and cause asymmetric loosening of DNA ends, inter-helical DNA bubbling adjacent to H4K79, and promote DNA sliding.

Overall, these all-atom computational modeling results lead to two discrete consequences. First, in comparison with CENP-A NCP, the rigidifying of the acetyl NCP locks the CENP-A C-terminus, stabilizes the H2A acidic patch, and disrupts CENP-C access to the CENP-A C-terminus. Second, asymmetric exposure of the DNA coupled with DNA bubbling may promote DNA sliding and thereby open the centromeric chromatin fiber to prime centromeric replication. To explore these possibilities in a cellular context, we next turned to an experimental approach in vivo.

### CENP-A K124 acetylation is not required for accurate targeting to centromeres

To dissect the function CENP-A K124 modifications in vivo, we used site-directed mutagenesis to generate versions of CENP-A K124 which mimic either a constitutively unacetylated state (Lysine to Alanine, K124A) or a constitutively acetylated state (lysine to glutamine, K124Q). These mutants were expressed in the endogenous CENP-A background and used for the series of experimental investigations discussed below (scheme in Additional file 4: Fig. S2A).

**Fig. 4:**
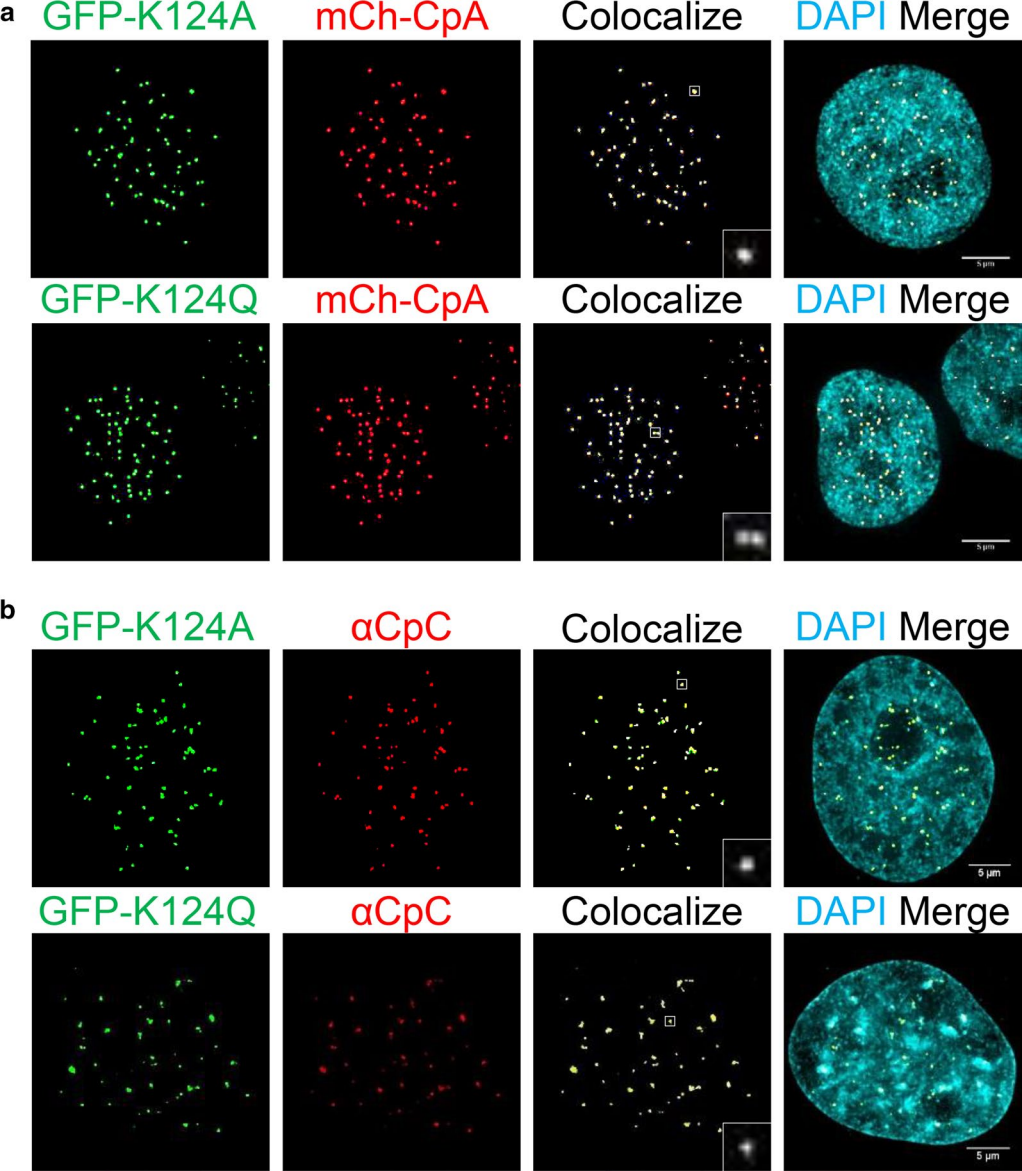
K124A/Q display normal centromeric localization. **a** CpA/K124A/K124Q were fused to an N-terminal GFP tag and co-expressed with mCh-CENP-A (CpA). The co-localization finder plug-in in ImageJ was used to find co-localizing GFP- and mCh-foci to indicate centromeric deposition, and final *panel* depicts a complete merge of all three channels including DAPI. **b** CENP-C (CpC) immunofluorescence (IF) was performed on GFP-tagged CpA/K124A/K124Q to look for co-localization. Puncta are *boxed* and magnified (in *white*) in the co-localize channel

We observed virtually no defects in centromeric localization for these mutants, as they overlap with mCh-CENP-A (Fig. 4a). To confirm this result, we co-immuno-stained K124A/Q along with the obligate inner kinetochore proteins CENP-C (Fig. 4b), and CENP-B (Additional file 5: Fig S3A-C), finding that these mutant foci also co-localized in a vast majority of the cells throughout the cell cycle. Although we did not measure the *efficiency* of assembly of mutants relative to endogenous CENP-A, these results suggest that CENP-A K124A and K124Q assemble at centromeres in a manner visually comparable to wild-type CENP-A.

### Constitutive gain or loss of K124 acetylation results in alterations in CENP-C binding

One key computational prediction from in silico experiments above (Figs. 1, 2, 3) was that acetyl CENP-A nucleosomes have reduced accessibility at the C-terminus, which results in reduced interactions with CENP-C. Therefore, we tested whether K124 acetylation alters interaction between modified CENP-As and CENP-C in vivo. We fused CENP-A (CpA), K124A, and K124Q to an N-terminal epitope HA-tag. This strategy avoids interference with the recognition motif for CENP-C on the CENP-A C-terminal tail [24, 27].

We first confirmed whether these mutant HA-CENP-A proteins were expressed normally in whole cell extracts (WCE). WCE for total protein revealed that exogenously expressed HA-tagged proteins, the CENP-A chaperone HJURP, and the inner kinetochore protein CENP-C levels remained relatively equal (Additional file 6: Fig. S4A).

**Fig. 5:**
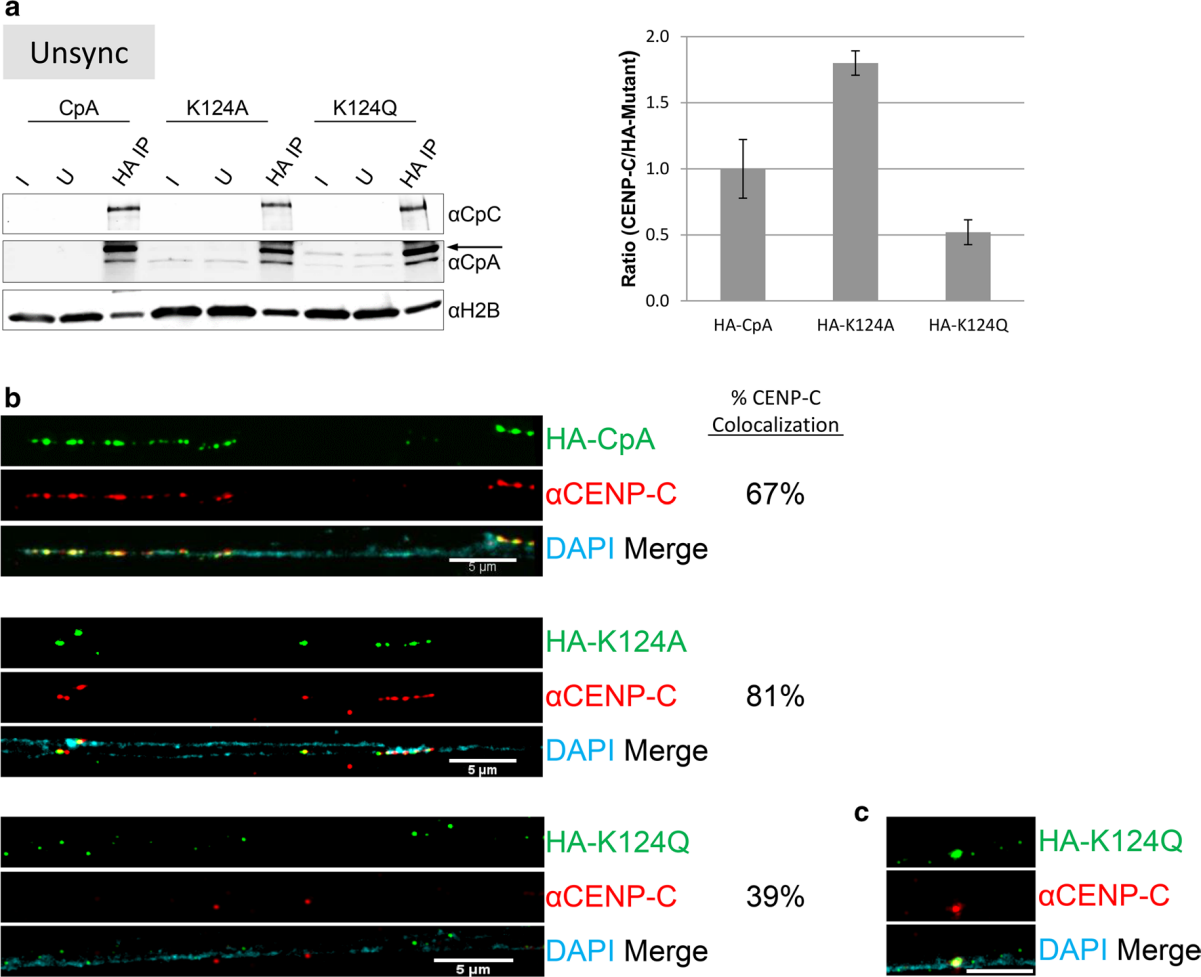
K124A/Q have altered affinity for CENP-C. **a** Anti-HA ChIP was performed on medium-sized chromatin arrays (see Additional file 6: Fig. S4D), and the amount of co-IP’ed CENP-C (CpC) was determined. *Arrow* indicates location of HA-tagged CpA/K124A/K124Q versus native CENP-A (nCENP-A) below. Three independent replicates conducted using HA-tagged CpA/K124A/K124Q mutants were analyzed by measuring the ratio of CENP-C/ChIP’ed HA-tagged mutant CpA/K124A/K124Q proteins, using LiCor Odyssey linear quantification software. *Bars* indicate standard error of the mean ratio. I = Input and U = Unbound. **b** Cells expressing HA-tagged CpA, K124A or K124Q mutants had their chromatin fibers extracted, and immuno-stained against HA (*green*) and CENP-C (*red*) to look for enrichment or depletion of CENP-C on the chromatin fiber (DAPI). Fibers with at least 50% of CENP-C co-localized to the CpA/K124A/K124Q foci were counted and percentage of co-localizing fibers determined. Distribution of the co-localizing fibers is in Additional file 6: Fig. S4C. **c** ‘Clumping’ K124Q interacts robustly with CENP-C on the chromatin fiber

HA-tagged CENP-As were then chromatin immunoprecipitated (ChIP’ed), 2 days after transfection, when mutant protein expression was at its highest and most detectable in WCE. Western blots were performed to detect the presence and levels of CENP-C relative to levels of CpA/K124A/K124Q (Fig. 5a; Additional file 6: Fig. S4B). Biochemical analysis also confirmed that endogenous CENP-A immunoprecipitates with CENP-C (Additional file 6: Fig. S4E). Next, we quantified the ratio of native CENP-C normalized against HA-tagged CpA/K124A/K124Q (Fig. 5a). Data from three independent experiments indicate that almost twice as much CENP-C binds unacetylatable K124A compared to CpA (Fig. 5a, graph). In contrast, the binding of acetyl-mimic K124Q to CENP-C is reduced by 50% relative to CpA (Fig. 5a, graph).

To verify this result using an alternative approach, we turned to co-immunofluorescence (co-IF) on chromatin fibers extracted from cells expressing either CpA, K124A, or K124Q. Next, we used anti-HA and anti-CENP-C antibodies to examine the association of CENP-C on CpA/K124A/K124Q positive chromatin fibers (Fig. 5b, Additional file 6: Fig S4C for detailed binning). We scored fibers as “co-localization positive” only if >50% spots on a given contiguous single DAPI-stained chromatin fiber showed co-localization of the HA-tagged mutant with CENP-C foci; conversely, if <50% of spots on a fiber did not co-localize, such fibers were scored as “co-localization negative.” Using this metric, we observed that 12/18 (67%) CpA fibers demonstrated robust co-localization with CENP-C. In contrast, an increase to 21/26 (81%) CENP-C-co-localized fibers were detected in the K124A mutants. However, K124Q fibers display two distinct behaviors, wherein the majority (61%) demonstrate no co-localization with CENP-C. However, a second population of fibers (11/28, or 39%) contains “clumped” K124Q foci, with co-localization between K124Q and CENP-C (Fig. 5c). Our interpretation of these data is that when CENP-A K124Q manages to fold into superfolded beads, these dense structures can stabilize CENP-C binding, potentially by direct or indirect associations (for example, if endogenous CENP-A is present within the superfolded structures), whereas the larger fraction of individual spots of CENP-A K124Q dotted along the same chromatin fiber do not retain CENP-C (Fig. 5b).

These chromatin fiber co-IF analyses support the biochemical results above, namely that relative to wild-type CpA, K124A has enhanced CENP-C associations, whereas K124Q has reduced associations with CENP-C.

### Constitutive gain or loss of K124 acetylation results in modest increase in mitotic errors

In previous experiments, over-expression of CENP-C in chicken DT40 cells led to higher rates of chromosomal segregation errors [29]. Since our data pointed to alterations in binding of CENP-C to mutant CENP-A, we were curious to test whether the presence of constitutive K124A or K124Q correlated with mitotic defects.

**Fig. 6:**
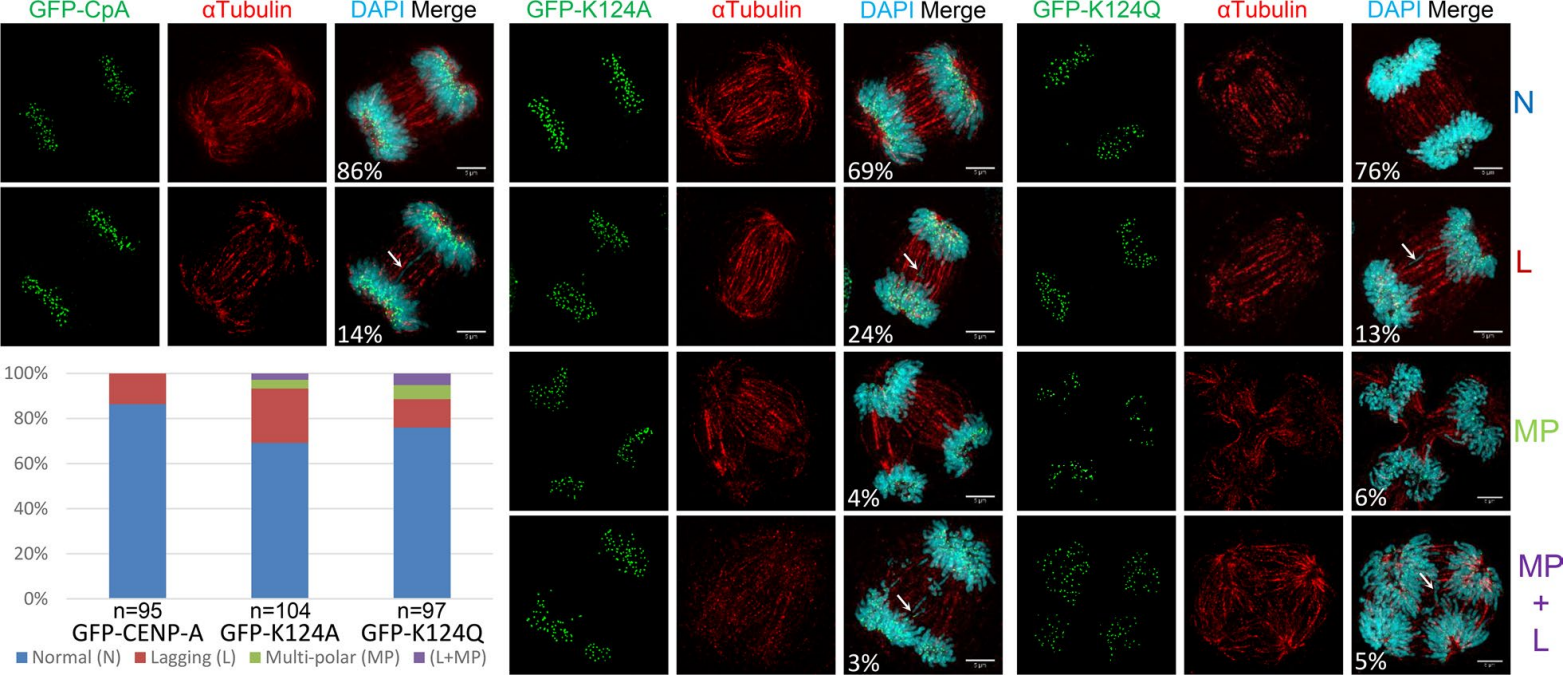
K124A/Q mutant cells have higher rates of mitotic defects compared to wild-type CENP-A. Cells were transfected with GFP-tagged CpA, K124A or K124Q, and synchronized with a double thymidine block to enrich for mitotic cells. Synchronized cells were immuno-stained for αTubulin, and images captured, binned and quantified into four categories: normal mitosis, mitosis with lagging chromosomes (*white arrow*), multipolar spindles, and multipolar spindles + lagging chromosomes (*white arrow*). Percentage of cells in each category for each mutant is shown graphically and values stated in the merge panel

Cells expressing CpA/K124A/K124Q were first synchronized with a double thymidine block. FACS analysis suggested that there were no appreciable defects or delays in cell cycle progression (Additional file 7: Fig S5). Next, these cells were immuno-stained with alpha tubulin, and anaphase cells scored to assess the frequency of multipolar spindles and lagging chromosomes. Consistent with previous data, GFP-tagged CENP-A (CpA) cells had 14% of cells with lagging chromosomes (white arrow) (Fig. 6). In contrast, K124A cells exhibited twice as many cells with defects, with 24% lagging, 4% multipolar, and 3% lagging + multipolar (Fig. 6). K124Q cells exhibited moderately increased number of cells with mitotic defects, with 13% lagging, 6% multipolar, and 5% lagging + multipolar (Fig. 6). Although we do not exclude that mitotic errors seen here arise from an accumulation of downstream defects, our data are consistent with previous observations that changes in CENP-C levels on CENP-A chromatin correlate with an increase in the rate of mitotic errors [29].

### K124A/Q have altered centromeric replication timing

Native CENP-A K124ac was first discovered during the G1 to S transition (G1/S), a critical junction before the onset of replication [20]. This led us to suggest that K124ac might contribute to centromere priming for replication [20, 30]. Indeed, prior work has demonstrated that a single modification within histone H3 sets the stage for accurate origin firing during replication [14, 31]. Furthermore, the computational predictions from the above experiments (Figs. 1, 2, 3) suggested that acetyl CENP-A nucleosomes had a higher potential for DNA unwrapping and sliding, which could promote CENP-A nucleosomal mobility. This in turn could provide the mechanistic basis for the findings that the centromeric chromatin fiber undergoes significant decompaction presaging the G1/S transition [20, 32]. To test whether long-term gain or loss of K124ac impacted centromeric replication, we returned our attention to S phase.

**Fig. 7:**
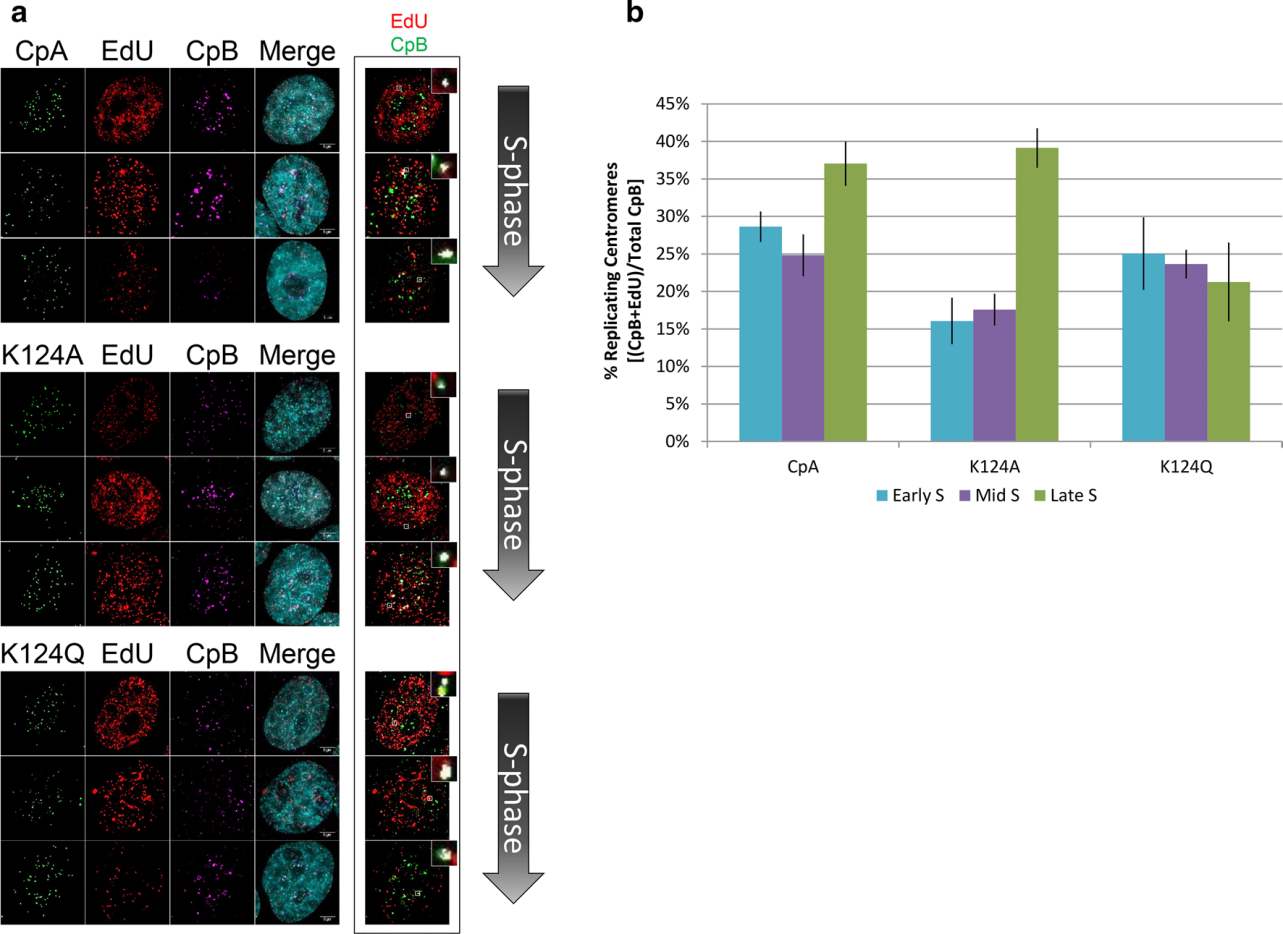
CENP-A mutations in K124 display altered replication timing of centromeric foci. **a** EdU-pulsed cells containing CpA/K124A/K124Q were co-stained with CENP-B (CpB) to assess percentage of centromeric replicating origins at early, mid and late S phases. 5 μm *scale bars* are located in the *bottom right* of the merge *panel*. *Boxed* to the *right* is an example of automated co-localization analysis using ImageJ, which was used to determine the fraction of co-localizing CpB and EdU foci (in *white*) with EdU +/CpB + *insets* to show co-localization. **b** A graphical representation of the percentage of centromeric origins co-stained with EdU (CpB + EdU) over the total number of centromeric (marked by CpB) foci

We synchronized cells to S phase and staged cells to early, mid- and late S phase time-points [33] (diagramed in Additional file 4: Fig. S2B). We used EdU staining to detect replication foci [34], coupled to CENP-B staining as a sequence-specific marker for human centromeric DNA. To quantify replication timing, we scored the number of EdU +/CENP-B + foci over the total number of CENP-B foci in CpA/K124A/K124Q expressing cells. We observed 25–30% EdU +/CENP-B + spots in CpA during early S and mid-S phases, with more of the EdU+/CENP-B+ co-localizing in late S phase (37%) (Fig. 7). These data are consistent with previous documentation of late replication of centromeres [35], with a minor fraction of centromere replication distributed throughout S phase [36, 37]. In contrast, K124A mutants had only 15% EdU+/CENP-B+ co-stained foci in early S and mid-S phases. By late S phase, replicating centromeres in the K124A mutant cells were restored to wild-type levels of 37%. These data suggest that the K124A mutant has a modest replication defect, in which it is slow to initiate centromeric replication in early S, but eventually restores to wild-type levels before the next mitosis.

Conversely, we observed that K124Q displayed a different phenotype. Whereas CpA shows a bias toward late S replication, K124Q mutants appear to have lost this bias, so that replicating centromeric origins fire equally throughout S phase (Fig. 7b). An alternative explanation is that because we are taking snapshots of cells captured at 2, 4, and 8 h after release from the double thymidine block, we could potentially miss an even earlier peak origin firing time for K124Q centromeres. Overall, these data suggest that constitutive gain or loss of K124 acetylation correlates with alterations in the distribution of centromeric replication timing.

### CENP-A K124 switches from acetylation at G1/S to monomethylation at S phase

**Fig. 8:**
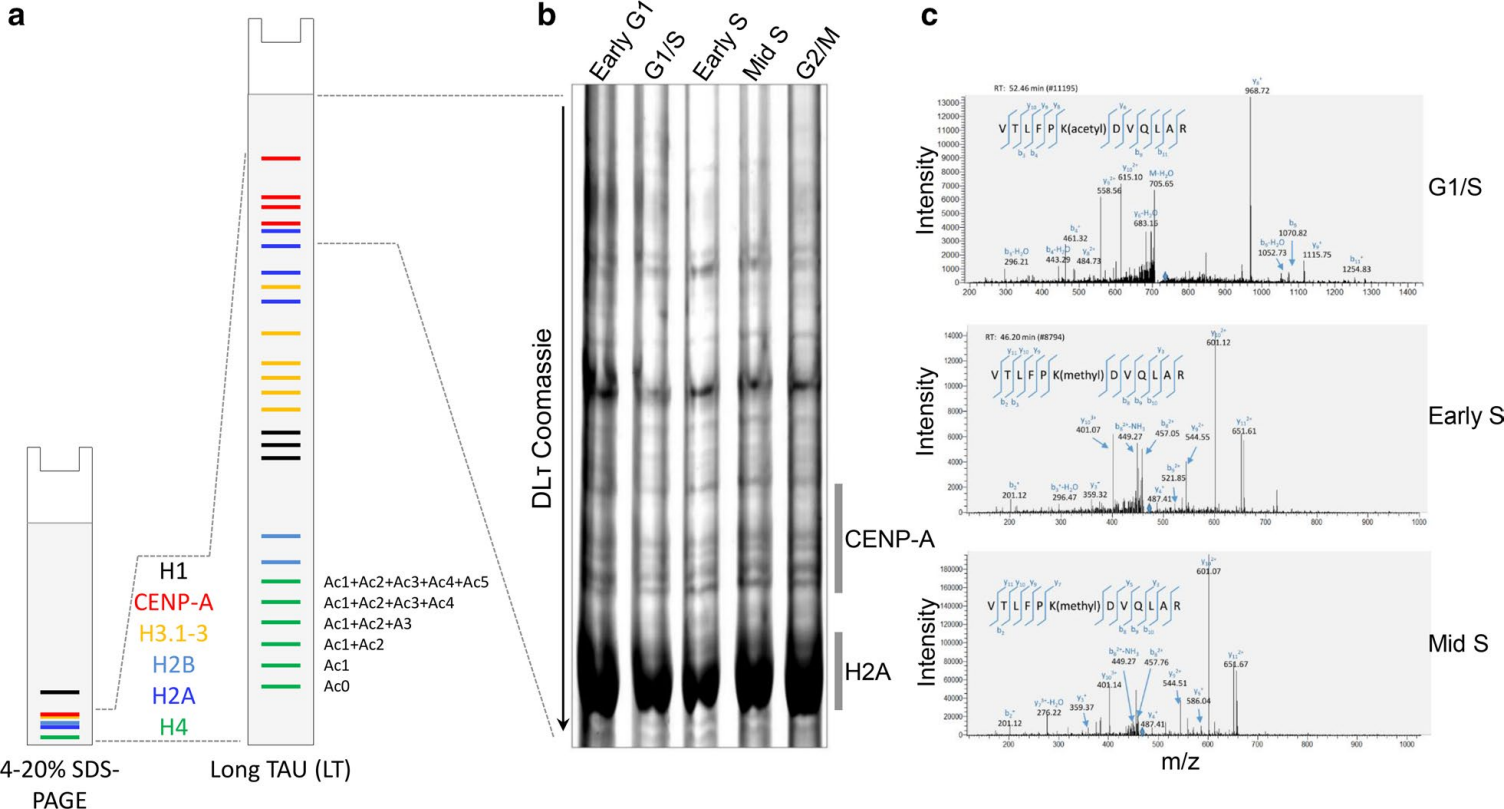
Endogenous CENP-A K124 is acetylated at G1/S but monomethylated at early S and mid-S phases. **a** Cartoon depicting the difference between resolving modified histones on a traditional SDS-PAGE versus a long TAU (LT) or double long TAU (DLT) gel. Each additional upward shift of the histone represents an additional acetylated residue. **b** G1/S, early S and mid-S chromatin-bound histones were isolated, resolved on a (DLT) gel, stained with Coomassie, and endogenous CENP-A species excised for subsequent analysis by mass spectrometry. **c** A peptide containing acetylated lysine 124 was observed in the G1/S sample. The representative MS/MS spectrum showing CENP-A K124 acetylated in the peptide “VTLFPK(acetyl)DVQLAR” is shown on the *bottom left*. Location of the parent peptide ion (*m*/*z* = 714.90, charge = +2) prior to fragmentation is indicated in each spectrum with a *blue diamond*. Peptide fragmentation ions identified are indicated in the spectra and on the peptide sequence. The masses of ions b9, b11, y8, y9, and y10 are diagnostic of K124 acetylation. The peptide containing monomethylated lysine 124 was observed in the early S and mid-S phase—the representative MS/MS spectrum showing CENP-A K124 methylated in the peptide “VTLFPK(methyl)DVQLAR” is shown on the *middle* and *top left*. Location of the parent peptide ion (*m*/*z* = 466.60, charge = +3) prior to fragmentation is indicated in each spectrum with a *blue diamond*. Peptide fragmentation ions identified are indicated in the spectra and on the peptide sequence. The masses of ions b8, b10, y7, y9 and y10 are diagnostic of K124 methylation

The replication defects noted above show that centromeric foci are modestly delayed in replication timing in K124A, and lack of a late S phase centromeric replication bias in K124Q cells. These phenotypes suggest that K124ac may be removed by early S phase, in order for centromeric replication to progress correctly. To address whether acetylation at K124ac is removed, or replaced by another modification at S phase, we utilized a native CENP-A gel-purification strategy coupled to a MS/MS approach [20, 38, 39, 40], adding a super-resolving, double long Triton-Acid-Urea (DLτ or DL-TAU) gel [41]. TAU gel chemistry is a robust method to study histone variants and their posttranslational modifications [38–40]. As histone residues become posttranslationally modified, such as the presence of a lysine acetylation, the positive to neutral charge of the residue induces a positive shift on the Lτ gel. As the schematic in Fig. 8a depicts, unlike traditional SDS-PAGE gels, an Lτ gel is sufficient to separate core histones into different species. For example, multiple histone H4 species were identified using TAU gels [42]. In our optimized version of this protocol (double long TAU, or DLτ), the majority of core histone proteins are electrophoresed off the gel, leaving behind only histone H2A at the bottom as a marker, resulting in distribution of CENP-A species throughout the middle of the gel (Fig. 8b, Additional file 8: Fig S6A). The corresponding CENP-A bands are excised and subjected to MS/MS (Additional file 8: Fig S6A), allowing enrichment of CENP-A species while reducing promiscuous contamination from H3 and H2A.

Mass spectra of CENP-A containing bands from these DLτ gels (Fig. 8b) confirm our earlier findings that CENP-A is acetylated at K124 during G1/S [20] (Fig. 8c, upper panel). Interestingly, this modification is not detected in either early S or mid-S phase (Fig. 8c, middle and lower panels). Instead, in both early S and mid-S phases, we detected robust methylation of K124 (Fig. 8c, middle and lower panels and Tables 1, 2 and 3, detailed list of spectra identified in MS/MS). Conversely, K124me was not detected in the G1/S CENP-A bands (Fig. 8c, upper panel). These data suggest a cyclical nature to modifications of K124, so that permanent gain or loss of acetylation at K124 in our mutants (Figs. 4, 5, 6, 7) likely disrupts the cycling of K124ac → K124me, which, in turn, impacts centromere dynamics during replication and subsequent mitoses.

**Table 1:** Mass spec fragment ion table for G1/S phase

#1	b^+^—theoretical	b^+^—experimental	b^2+^—theoretical	b^2+^—experimental	Seq.	y^+^—theoretical	y^+^—experimental	y^2+^—	y^2+^—experimental	#2
1	100.0757		50.54149		V					12
2	201.12338		101.06533		T			665.37993	665.66638	11
3	314.20745	314.2298	157.60736		L	1228.7049	1228.83008	614.85609	615.0849	10
4	461.27587	461.30969	231.14157		F	1115.62083	1115.7052	558.31405	558.50128	9
5	558.32864	558.50128	279.66796		P	968.55241	968.66461	484.77984	485.78	8
6	728.43417		364.72072		K-Acetyl	871.49964	871.6875	436.25346		7
7	843.46112	843.41919	422.2342		D	701.3941	701.29291	351.20069		6
8	942.52954	942.66	471.76841	471.3	V	586.36715	586.47998	293.68721		5
9	1070.58812	1070.74	535.7977		Q	487.29873	487.38043	244.153		4
10	1183.67219	1183.84912	592.33973	592.63	L	359.24015	359.27	180.12371		3
11	1254.70931	1254.79114	627.85829	627.32	A	246.15608	246.14738	123.58168		2
12					R			88.06312		1

**Table 2:** Mass spec fragment ion table for early S phase

#1	b^+^—theoretical	b^+^—experimental	b^2+^—theoretical	b^2+^—experimental	b^3+^—theoretical	b^3+^—experimental	Seq.	y^+^—theoretical	y^+^——experimental	y^2+^—theoretical	y^2+^—experimental	y^3+^—theoretical	y^3+^—experimental	#2
1	100.08		50.54		34.03		V							12
2	201.12	201.13	101.07		67.71		T	1301.75766		651.38247	651.58466	434.59074	434.88	11
3	314.21	314.26	157.61		105.41		L	1200.70998		600.85863	601.03448	400.90818	401.00195	10
4	461.28	460.65	231.14		154.43		F	1087.62591		544.31659	544.31	363.21349	363.26254	9
5	558.33	558.50	279.67	279.13	186.78		P	940.55749		470.78238		314.19068	314.26239	8
6	700.44		350.72	350.18	234.15		K-Methyl	843.50472		422.256	422.16428	281.83976	281.40979	7
7	815.47		408.24	408.10	272.49	272.21	D	701.3941		351.20069	351.17957	234.46955		6
8	914.53		457.77	458.04	305.52		V	586.36715	585.92181	293.68721	294.14	196.12723		5
9	1042.59		521.80	521.63	348.20		Q	487.29873	487.35	244.153	244.11246	163.10443		4
10	1155.68		578.34	578.60	385.90	385.20	L	359.24015	359.29077	180.12371		120.41823		3
11	1226.71		613.86	613.82	409.58	409.25	A	246.15608	246.22052	123.58168		82.72354		2
12							R	175.11896	175.16	88.06312		59.0445		1

**Table 3:** Mass spec fragment ion table for mid-S phase

#1	b^+^—theoretical	b^+^—experimental	b^2+^—theoretical	b^2+^—experimental	b^3+^—theoretical	b^3+^—experimental	Seq.	y^+^—theoretical	y^+^—experimental	y^2+^—theoretical	y^2+^—experimental	y^3+^—theoretical	y^3+^—experimental
1	100.08		50.54		34.03		V						
2	201.12	201.16	101.07		67.71		T	1301.76		651.38	651.67	434.59	434.74
3	314.21		157.61		105.41		L	1200.71		600.86	601.07	400.91	401.14
4	461.28	460.52	231.14		154.43		F	1087.63		544.32	544.51	363.21	
5	558.33		279.67		186.78		P	940.56		470.78		314.19	
6	700.44		350.72		234.15		K-Methyl	843.50		422.26		281.84	
7	815.47		408.24		272.49		D	701.39		351.20		234.47	
8	914.53		457.77	457.76	305.52		V	586.37	586.04	293.69		196.13	
9	1042.59		521.80	521.75	348.20		Q	487.30	487.34	244.15		163.10	
10	1155.68		578.34		385.90		L	359.24	359.37	180.12		120.42	
11	1226.71		613.86		409.58		A	246.16		123.58		82.72	
12							R	175.12		88.06		59.04	

### HATs and HDACs influencing the acetylation of native CENP-A K124

To investigate HATs and HDACs which influence modification of K124 in endogenous CENP-A, we turned again to Lτ gels coupled to WBs. Many commercially available and custom CENP-A antibodies cross-react with the far more abundant and heavily modified H3 (*unpublished observations*), which co-migrates with CENP-A on standard SDS-PAGE gels. To avoid this problem, we couple TAU gels to WBs for studying CENP-A modifications, where H3 and CENP-A run far apart, avoiding potential cross-reactivity. We developed a custom rabbit polyclonal antibody against CENP-A K124ac. Using recombinant histone proteins including CENP-A, H4, synthetic CENP-A K124ac, H4K79ac, H2A and H2B, we first tested this polyclonal antibody (Additional file 8: Fig. S6B) on TAU-WBs, finding that it has high specificity for the K124ac species.

Next, we examined the role of various HAT/HDAC complexes on CENP-A K124ac. We treated cells with established histone acetyltransferase (HAT) and histone deacetylase (HDAC) inhibitors. C646 is a HAT inhibitor that targets p300 (HATi^p300^), trichostatin A (TSA) is a potent pan HDAC inhibitor (HDACi^PAN^), and quisinostat 2HCl is a pan inhibitor that targets predominantly HDAC1, 2, 4 and 10 (HDACi^1,2,4,10^). P300 was predicted to be a HAT target for CENP-A, because of a previous study, in which co-localization of p300 with CENP-A was observed at centromeres [43]. Furthermore, it has been shown that tethering of a p300 catalytic domain induces assembly of CENP-A onto alphoid DNA [44, 45], in which it was suggested that acetylation of CENP-A was required for its stable retention on chromatin.

**Fig. 9:**
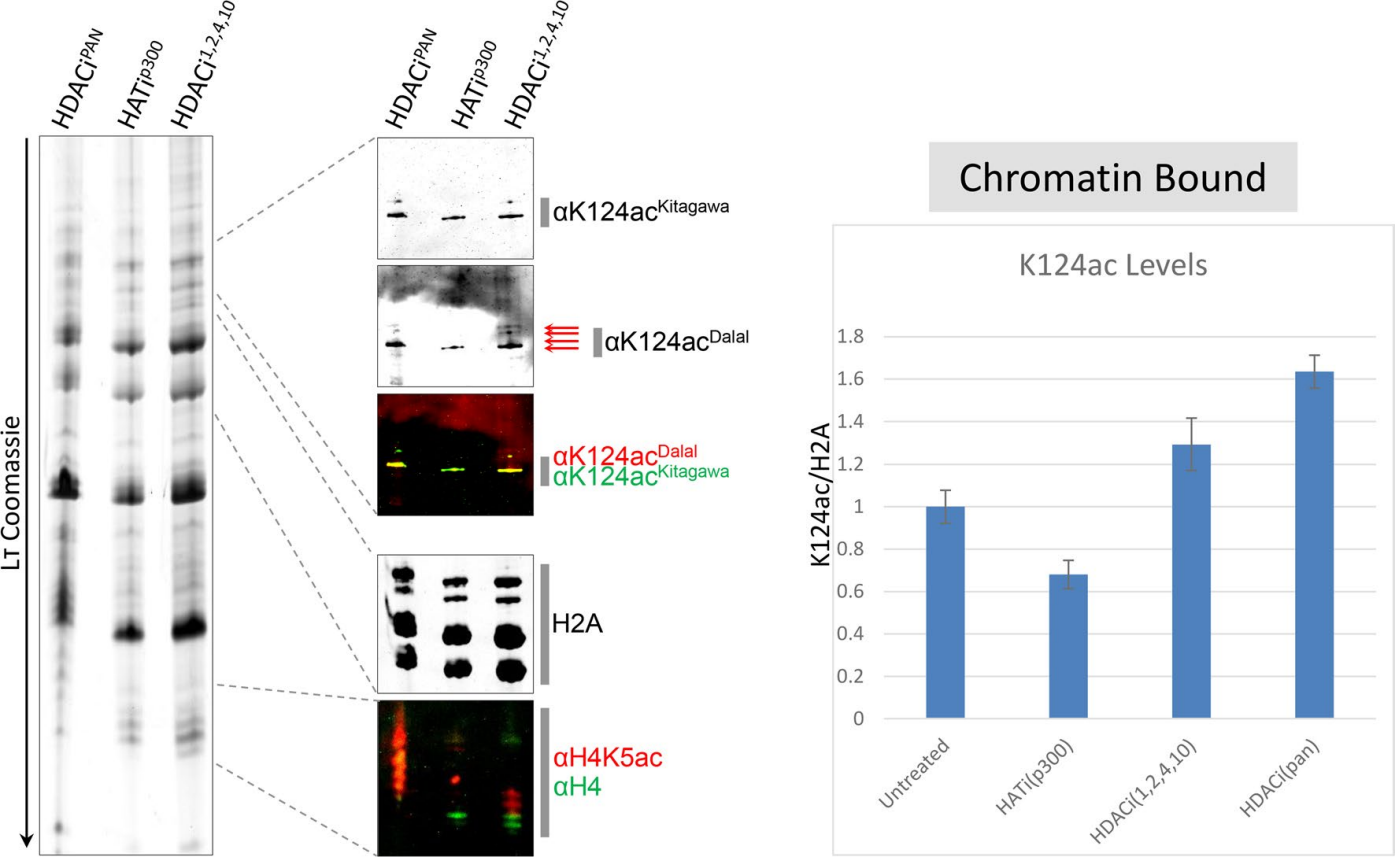
Histone acetyltransferase p300 targets CENP-A K124 for acetylation. Cells were treated for 20 h with TSA (pan HDAC inhibitor = HDACi^PAN^), C646 (HAT inhibitor against p300 = HATi^p300^) or a weak pan HDAC inhibitor that targets HDAC 1, 2, 4 and 10 (HDACi^1,2,4,10^). Chromatin-bound histones were purified from nuclei and ran on an LT gel followed by Western analysis. Average K124ac levels were normalized against total histone H2A and plotted relative to untreated = 1. Whole cells extracts and recombinant unmodified CENP-A and CENP-A K124ac proteins were also loaded on an LT gel, followed by Western analysis to determine antibody specificity (please refer to Additional file 8: Fig. S6B)

Cells were untreated with control DMSO (Additional file 8: Fig. S6C), or DMSO with 20 µM C646 or 200 nM TSA, for 20 h, harvested, and subsequent purification of either whole cell extracts (WCE) (Additional file 8: Fig. S6B), or chromatin-bound histones using hydroxyapatite elution [20] (Fig. 9). In both cases, WCE or chromatin-bound, histones were probed with the CENP-A K124ac antibody, CENP-A, H2A and H4. As a control, we examined H4K5ac levels, because they should be reduced when the HAT p300 is inhibited by C646 [46]. As expected, H4K5ac levels diminished significantly in the p300 inhibitor (C646) treatment compared to untreated, suggesting effective inhibition of p300 (Additional file 8: Fig. S6B, Fig. 9, H4K5ac panel). We first examined WCE, in which, after normalizing CENP-A K124ac relative to total histones on the TAU gel, p300 inhibition did not impact CENP-A K124ac levels (Additional file 8: Fig. S6B). We next interrogated chromatin-bound histones, relative to histone H2A as a loading and normalizing control over untreated. As expected, in the p300 inhibition lane, control H4K5ac levels were significantly reduced. We next co-stained our TAU-WBs with our custom K124ac antibody, along with a second antibody (gift, Kitagawa laboratory), which also recognizes CENP-A K124ac. Reassuringly, both K124ac antibodies recognized the same CENP-A species on TAU-WB (Fig. 9). Unlike in WCE, chromatin-bound levels of CENP-A K124ac in the HAT- and HDAC-inhibited lanes were markedly different than the untreated sample (Fig. 9, Additional file 8: Fig. S6C). CENP-A K124ac levels were reduced by about 40% (compared to untreated) in the p300 inhibited cells, while there was a 30 and 60% increase in the quisinostat and pan HDAC TSA treatments, respectively (Fig. 9). These data suggest that p300 likely functions in the K124 acetylation pathway. Our evidence also supports the idea that multiple HDACs may be involved in removing the acetylation of K124, because we observed reduction of K124ac species in both pan and HDAC-specific inhibitor treatments (Fig. 9). Taken together, these data suggest p300 functions on chromatin-bound CENP-A to promote its acetylation.

## Discussion

Despite decades of intensive biochemical studies, precisely how histone modifications impact biological function has been difficult to dissect. In recent years, elegant in vivo and in vitro experiments demonstrate that core modifications can exert effects on nucleosome behavior, chromatin changes, and biological function [6, 9, 10, 47, 48]. In previous work, we reported that during G1/S, human centromeric chromatin becomes more accessible and that CENP-A nucleosomes occupy a transitionary state concurrent with internal histone fold domain acetylations at CENP-A K124 and H4K79 [20]. In this work, we probed the effects of CENP-A K124 acetylation using an interdisciplinary approach.

Computational modeling demonstrates that CENP-A K124ac and H4K79ac collaborate to loosen the DNA at four symmetric contact points within the nucleosome, which is predicted to promote access to the nucleosomal DNA while simultaneously rigidifying the protein core. In silico results also suggest that dual acetylation of CENP-A K124 and H4K79 serves an un-anticipated role in restraining the C-terminus of CENP-A so that it is less accessible than in the unmodified CENP-A NCP (Figs. 1, 2, 3). One outcome of this “locking” of the CENP-A C-terminus is a reduced binding to CENP-C in silico.

In subsequent biological experiments, we expressed mimics of acetylated K124 (K124Q) or unacetylatable K124 (K124A) CENP-A. Although we did not observe any localization defects, we did note that CENP-A K124A mutant is expressed poorly compared to the CpA/K124Q mutant (Additional file 6: Fig. S4B) 2 days after transfection. These data could be interpreted to suggest that that K124A is either poorly deposited, or that excess un-incorporated K124A is degraded more rapidly relative to wild-type. This interpretation would partially support recent work showing that the ubiquitination of K124 at early G1 promotes centromeric deposition of CENP-A [22].

In testing the hypothesis that CENP-C binding is altered, we noticed that both K124A/K124Q mutants have altered CENP-C binding (Fig. 5). In chromatin-IP-coupled Western blots, we observed that CENP-C co-associated more strongly with K124A than with wild-type and that the K124Q-acetylated mimic displayed a reduction in CENP-C. These results are supported by chromatin fiber co-IF, which likewise shows a reduced association of CENP-C on K124Q fibers, and a relatively increased abundance on K124A fibers. Consistent with direct or downstream accumulated changes in CENP-C binding, K124A and K124Q cells display a relative increase in mitotic defects relative to wild-type CENP-A cells (Fig. 6). These data are consistent with earlier experiments, documenting that titration of CENP-C levels lead to higher mitotic error rates [29].

Our experiments investigating the role of K124ac at replication show that in K124Q cells, the normal late S phase centromere replication bias is lost, whereas in K124A cells, centromeric replication is delayed from early S to mid-S phase (Fig. 7). The mechanistic basis for this observation remains under investigation. The simplest possibility is that decompaction of the CENP-A chromatin fiber is a prerequisite to its replication. In vitro studies of unmodified CENP-A on 601 DNA sequences [49, 50] have reported that CENP-A chromatin fibers are more compacted. We speculate that G1/S-specific CENP-A K124ac/H4K79ac promotes unfolding of the centromeric fiber, possibly by increased nucleosomal sliding, and transient release of CENP-C from the CENP-A C-terminus, possibly repositioning CENP-C to the intervening linker DNA. Recent work shows that centromeric replication timing is exquisitely sensitive to the state of the chromatin fiber. The pre-replication complex (pre-RC) consists of ORC1 through ORC6 [51], of which only ORC2 has been shown to specifically associate with centromeres at S phase [31]. Depletion of the key inner kinetochore protein CENP-B alters the chromatin landscape at centromeres and enhances the binding of ORC2 to centromeric origins [37]. Thus, the centromeric replication defects observed in our study above (Fig. 7) might be underpinned molecularly by a combination of chromatin fiber accessibility and mis-timing of the interaction between K124A/Q mutant proteins with pre-RC components. Importantly, the defects in replication timing of the K124Q acetylated mimic point to the importance of *removing* the acetylation. Here, our TAU-MS/MS data demonstrate that CENP-A K124 acetylation is replaced by monomethylation at S phase (Fig. 8).

Precedence for histone core domain methylation in regulating replication also exists. Methylation of H3K79 limits genome-wide DNA replication to once per cell cycle, thereby preventing over-replication [52]. In our mutants, neither K124A nor K124Q can undergo methylation, breaking the potential cycle of K124ac to K124me in S phase (Additional file 9: Fig. S7A-B). We hypothesize that one putative function of K124me may be to stabilize the nucleosomal DNA, either by rapid re-binding of CENP-C immediately after replication, or by inhibiting re-binding of pre-RC components to the newly replicated CENP-A chromatin, thereby preventing over-replication (Additional file 9: Fig. S7C). Additional future experiments that are necessary include dissecting how HATs like p300, which, as shown above (Fig. 9) contributes to CENP-A′s acetylation, are recruited to the centromeric fiber, and identifying the HMT responsible for methylating CENP-A K124. Excitingly, ongoing in vitro dissections using synthetically engineered CENP-A K124ac [53] coupled to H4K79ac proteins, will determine whether acetylated CENP-A nucleosomes encode a chromatin fiber more susceptible to sliding, and whether such a fiber has an altered affinity for CENP-C.

## Conclusions

In this report, we dissect the role of a single modification in the CENP-A nucleosome in silico and in vivo, finding it to be involved in regulating CENP-C distribution on the modified CENP-A chromatin fiber, mitotic integrity, and centromere replication timing. We emphasize that the delays in centromere replication timing we observed are not permanent, as most cells recover by late S phase. Indeed, majority of the mutant cells survive in the presence of K124A and K124Q, and, despite the small but measurable increase in mitotic defects, we did not observe persistent deleterious effects. Thus, while acetylation of CENP-A K124 does not appear to play a deterministic role, it does subtly contribute to centromere dynamics at the level of replication timing and mitotic integrity. Consequently, these data open a new avenue of investigation into how covalent modifications, buried within the histone fold domain of histone variant nucleosomes, can serve as epigenetic regulators of biological processes.

## Methods

### Simulation protocol

All-atom molecular dynamics (MD) simulations were performed with software suite Gromacs 5.0.4 [54]. The force field employed to model nucleosomes was amber99SB*-ILDN [55, 56] for proteins, amber99SB parmbsc0 [57] for DNA, ions08 [58] for ions, and the TIP3P [59] water model.

Two nucleosomal systems were built for simulation: the acetyl-lysine CENP-A nucleosome and then unmodified CENP-A nucleosome as control. First, the CENP-A nucleosome was built with PDB ID: 3AN2^1^—resolved to 3.60 Å—as the starting structure. Unresolved 3AN2 residues Thr 79 through Asp 83 of CENP-A′, Chain E, were built with MODELLER [60]. During energy minimization of this constructed region, one residue in the n-terminus and c-terminus directions was unconstrained. Additionally, heterogen selenomethionine residues were altered to methionine through a single-atom mutation from Se to S. As a control, the 146 base-pair α-satellite DNA of PDB ID: 3WTP [61] was aligned onto 3AN2 using the CE algorithm [62] of PyMOL [63]. To further study the effect of acetylation on CENP-C binding, the CENP-C fragment [27] was docked onto each system using the CE algorithm [62] and simulated for an additional microsecond retaining identical simulation protocols. This is the first known all-atom detail structural analysis of CENP-C bound to CENP-A.

From this initial structure, the Gromacs tool pdb2gmx was used to assign charges to residues at biological pH: a charge of +1 on Lys and Arg, 0 for Gln, −1 for Asp and Glu, and His with hydrogen on the epsilon nitrogen. Then, a rectangular cuboid box was created such that boundaries were a minimum distance of 1.5 nm from the unsolvated system. Next, Na+ and Cl− ions were introduced to neutralize the system charge and additionally model an ionic concentration to 150 mM. For both pre-production and production runs, periodic boundary conditions were employed. Electrostatics were handled with the particle-mesh Ewald method and Verlet cutoff scheme. For the non-bonded interaction shift functions, Coulombic and van der Waals potentials had a cutoff distance at 1.0 nm. Hydrogen bonds were constrained with the LINCS algorithm.

The CENP-A nucleosome system was energy minimized using steepest descent to a maximum energy of 100 kJ/mol. The systems were then equilibrated in multiple steps. First, the systems were heated to 300 K for 2000 ps. During this step, DNA was restrained with *K* = 1000 kJ mol^−1^ nm^−2^ in the canonical ensemble (NVT). For the next thermal equilibration at 300 K for 2000 ps, both DNA and protein had weak harmonic position restraints, *K*_CENP-A_ = 2.5e−5 kJ mol^−1^ nm^−2^, to hinder global rotational motions. Lastly, pressure was equilibrated for 1500 ps in the isothermal isobaric, NPT, ensemble at 300 K and 1.0 bar with *K*_CENP-A_.

This system was ran for 1 μs at 300 K. Temperatures were V-rescaled with the modified Berendsen thermostat [64] with a time constant of 1.0 ps. System pressures were regulated with the Parrinello–Rahman barostat [65] at 1.0 bar and a time constant of 2.0 ps. The simulations’ time step size was 2 fs. Coordinates, velocities, and energies were saved every 2 ps. Non-bonded neighbors lists were updated every 20 fs.

After the CENP-A nucleosome was run for 1 μs, the final structure was acetylated in four histone core locations: K124 of CENP-A and CENP-A′, and K79 of H4 and H4′. The partial charges assigned to acetyl-lysine atoms were calculated quantum mechanically as described previously [66]. The new amino acid type for acetyl-lysine, KAC, was added to amber99SB*-ILDN^2,3^. Both the acetyl-lysine CENP-A system and the control CENP-A system were simulated for an additional 1 μs as described above. For subsequent analysis, trajectories were truncated to remove the first 600 ns to account for additional system equilibration during production runs.

### Analysis of trajectories

After truncating the simulation data to the final 400 ns for analysis, the root-mean-square fluctuations (RMSF) were calculated for the Cα atoms of the histones and the average of the nucleic acids for DNA. The RMSF is used to calculate local time-averaged fluctuations. The RMSF of DNA (Additional file 2: Fig. S1) was calculated for thirds of the final 400 ns and then the standard deviation of the mean plotted. Contact analysis was calculated with a cutoff distance of 8 Å between histone Cα atoms to compare dimer interface contacts in both systems. The center of mass (COM) of dimers was then calculated along the trajectory and the distribution of distances between COMs compared.

Principle component analysis (PCA) was performed on the histone core as previously described^2^. This analysis was then extended to include Cα atoms of histones and phosphate atoms nucleosomal DNA. The first and last ten base pairs were truncated from the analysis to remove ends calculated to have a high RMSF (Additional file 2: Fig. S1). This alteration was made so that DNA end motions did not dominate the major principle components. The magnitude of motion is multiplied by a factor of 5 in the movies to amplify motions for visual clarity.

### Cloning

GFP-CENP-A plasmids were a gift from Stephan Diekmann. To mutate K124 to alanine (A) or glutamine (Q) residues, fusion PCR was performed using a reverse primer (TGGGAAGAGAGTAACTCGG) along with a forward primer from the 5′ START codon that includes an EcoRI site. That amplicon was gel purified and combined with a PCR amplicon that used a forward primer encompassing the K to [A] or [Q] mutation (CGAGTTACTCTCTTCCCA[GCG]GATG or CGAGTTACTCTCTTCCCA[CAG]GATG, respectively) and a reverse primer that includes the XmaI site and STOP codon. The final fusion PCR product was excised, gel purified and finally ligated downstream of plasmid that had either GFP or HA-tags, driven by a constitutive CMV promoter.

### Transfections

HeLa cells were grown to ~75% confluency and transfected using Lonza’s Amaxa Cell Line Nucleofector Kit R (Cat #VCA-1001) using Amaxa Biosystems Nucleofector II electroporation system according to the manufacturer’s guidelines using program O-005. After transfection, cells were plated with fresh media and grown for 48 h before harvesting for experiments.

### Cell synchronization, native chromatin immunoprecipitation (nChIP)

HeLa cells were grown in DMEM (Invitrogen/ThermoFisher Cat #11965) supplemented with 10% FBS and 1X Pen/Strep. nChIP experiments were performed without fixation, and with or without a double thymidine block to synchronize cells. For the complete double thymidine block protocol, please refer to Bui et al. (2012).

After cells were harvested, they were washed with PBS and PBS containing 0.1% Tween 20. Nuclei were released with TM2 (20 mM Tris–HCl, pH: 8.0; 2 mM MgCl_2_) with 0.5% Nonidet P 40 Substitute (Sigma Cat #74385). Afterward, nuclei were washed with TM2 and chromatin was either digested for 4 min for nChIP or 8 min for ChIP-seq with 1.0 U MNase (Sigma Cat #N3755-500UN) in nuclei solubilized with 2 mL of 0.1 M TE (10 mM Tris, 0.2 mM EDTA, 100 mM NaCl) and supplemented with 1.5 mM CaCl_2_. MNase reactions were quenched with 10 mM EGTA and centrifuged at 1000 rpm at 4 °C. Supernatant was removed, and nuclei extracted overnight at 4 °C in 0.5X PBS supplemented with a protease inhibitor cocktail (Roche Cat #05056489001). ChIP was performed with anti-HA antibody (Santa Cruz Cat #sc-805). nChIP’ed chromatin bound to Protein G Sepharose beads (GE Healthcare Cat #17-0618-02) were washed 3X with cold 0.5X PBS. Westerns were done using LiCor’s Odyssey CLx scanner and Image Studio Ver 2.0. Antibodies used for Westerns include: CENP-A (AbCam Cat #ab13939), CENP-B (AbCam Cat #ab25734, CENP-C (MBL Cat #PD030), HA-tag (GenScript Cat #A01244), FLAG-tag (AbCam Cat #ab1162), and H2B (AbCam Cat#52484).

### TAU gels and Westerns

For synthesis, preparatory and running conditions for TAU gels, please refer to Walkiewicz, Bui [67]. For Western transfers, we used the Trans-blot Turbo Transfer Pack (mini Biorad Cat #170-4158 or midi Biorad Cat #170-4159). For Western detection of the proteins, we used LiCor’s secondary infrared antibodies, the Odyssey CLx laser scanning system, and Image Studio Ver 2.0 to quantify the protein levels.

### Immunofluorescence (IF)

For complete cell and chromatin fiber IF protocols, please refer to Bui et al. (2012). GFP tags were fused in-frame and upstream of wild-type CpA, K124A or K124Q, and may be co-transfected with mCh-CENP-A for co-localization assays. IF was performed using CENP-B (AbCam Cat #ab25734) and CENP-C (MBL Cat #PD030) antibodies. Cells were pulsed with EdU 30 min prior to the desired time point using the Click-iT EdU Alexa Fluor 594 kit (Life Technologies Cat #C10639) and imaged using a DeltaVision RT system fitted with a CoolSnap charged-coupled device camera and mounted on an Olympus IX70. Deconvolved IF images were processed using Image J and to assess co-localization with its ‘Colocalization Finder’ plug-in.

### LC–MS

Tau gel bands were processed using an in-gel digestion protocol from Shevchenko et al. (*Nature Protocols* **1**, 2856–2860, 2007). Each band was split in two, and separate trypsin and chymotrypsin in-gel digestions were performed. The samples then underwent shotgun proteomic analysis on a nano-HPLC system (NanoLC 2D; Eksigent, Dublin, CA) coupled to a hybrid mass spectrometer (Orbitrap Velos Pro; Thermo-Electron, Bremen, Germany). Samples were injected using an auto-sampler and loaded onto a self-packed trap column (2 cm, 100 µm ID, packed with C18 Magic AQ from Michrom Bioresources, Auburn, CA), and the samples were then analyzed on a self-packed C18 (15 cm, 2.7 µm HALO Peptide ES C-18, MAC-MOD, Chadds Ford, PA) column with a laser-pulled tip (P-2000, Sutter, Novato, CA) using a flow rate of 200nL/min. The column was heated to 50 °C using column heater (Phoenix S&T, Chester, PA). Mobile phase A was water with 0.1% formic acid, and mobile phase B was acetonitrile with 0.1% formic acid. The analytical gradient was a 90-min linear gradient from 5 to 35% buffer B. Eluting peptides were electrosprayed at 2.3 kV, and the ion transfer capillary was heated to 250 °C. The Orbitrap was operated in data-dependent mode with different settings depending on the cleavage enzyme used: Trypsin-cleaved samples were analyzed with a CID top 18 method, and chymotrypsin-cleaved samples were analyzed with a CID and ETD decision tree top 12 method. Precursor resolution was set to 60,000, CID collision energy was 35%, and ETD time reaction time was 100 ms with supplemental activation.

### Database search parameters

Protein identification was performed against the UniProt database entry for CENP-A using Proteome Discoverer 2.1 (Thermo Fisher Scientific) equipped with SEQUEST HT and Mascot (Matrix Science, Boston, MA). Search settings included tryptic or chymotryptic digest with up to two missed cleavages or nonspecific cleavage. Carbamidomethylation of cysteine was set as a static modification, while dynamic modifications included Met oxidation, Asp, Glu deamidation, Ser, Thr, Tyr phosphorylation, Lys acetylation, Lys ubiquitination, Arg and Lys methylation. Only matches with XCorrs greater than 2.0 or ion scores greater than 20 were considered. All the spectra matches were manually validated.

## Additional files

**Additional file 1: Movie S1.** Histone dimers CENP-A/H4 is shown in red, CENP-A′/H4′ in blue, H2A/H2B in light blue, and H2A′/H2B′ in white. Movies presented here are animations of the most significant mode of motion, PC1^core^, of the principal component analysis of histones (PCA^core^). The first clip shows the histones in both systems rocking with a “freezing” of motion in the acetyl CENP-A histones on the right. The second clip shows the 4-helix bundle in isolation to highlight the interface formed between CENP-A and CENP-A′. Next, the histones are rotated to focus on the described scissoring motion between helices α2 and α3 in the acetyl NCP. Here, the two helices move apart and then together, modulating the widths of the major and minor grooves in the acetyl NCP. Next, flipped to the other side of the nucleosome core, observe the rigidification of the H2A/H2B to H2A′/H2B′ interface in the acetylated nucleosome. To clarify this further, we then show the H2A to H2A′ interface in isolation.

**Additional file 2: Fig. S1.** RMSF of proteins. **A**) This decrease in RMSF of Cα residues upon acetylation is more pronounced on the histone heterotetramer adjacent to the entry DNA. Of particular interest, the RMSF of the acetylated H2A acidic patch was suppressed with acetylation by −1 Å, and suppression is shown in the CENP-A C-terminus. The greater similarity shown in the RMSF of the reciprocal histones—CENP-A′, H4′. H2A′, and H2B′—could potentially be explained by the observed asymmetric unwrapping of DNA where the exit end in both systems dissociates to a similar amount (**Fig.** **3A**). **B**) The RMSF of whole base pairs is shown for each DNA strand. Regions marked by I are DNA wrapped near the entry or near CENP-A and II are near the exit end of CENP-A′. The pseudo-dyad is marked by the vertical dotted line.

**Additional file 3: Movie S2.** Histone dimers CENP-A/H4 is shown in red, CENP-A′/H4′ in blue, H2A/H2B in light blue, and H2A′/H2B′ in white. Presented are animations of the most significant mode of motion of the whole nucleosome, PC1^nuc^, of the principal component analysis (PCA^nuc^). The pseudo-dyad is labeled PD, and the modified lysine side chains are shown in green with or without acetylation dependent on the system. It is worth noting that our PCA^nuc^ calculations are based on DNA phosphate positions and protein Cα’s—therefore, side chains are stagnant relative to the protein backbone. In the first two clips, two unique features of the acetyl NCP are shown: the modulation in the width of the major and minor DNA grooves and the inter-helical DNA bubble formed adjacent to H4 and H4′ K79ac. The final clip then shows the NCP on the side to highlight DNA end untwisting in the acetyl NCP with the last ten base pairs were truncated from the analysis.

**Additional file 4: Fig. S2.** Experimental scheme for experiments and cell synchronization. **A**) Computation and biological experimental scheme for this publication. **B**) Cell cycle synchronization with a 30-min EdU pulse prior to preparing slides for EdU and immunofluorescence.

**Additional file 5: Fig. S3.** Co-CENP-B/CENP-C staining with HA-tagged K124A/Q proteins. **A**) Unsynchronized cells stained with CENP-B (CpB) and **B**) percentage of CENP-B co-localizing with GFP-CENP-A (CpA) or K124A/Q exogenously expressed proteins after a double thymidine block and released for 1 or 8 h. **C**) Co-localization of the GFP-tagged CpA/K124A/K124Q with CpB during metaphase.

**Additional file 6: Fig. S4.** K124A/Q have altered affinity for CENP-C. **A**) Whole cell extracts (WCE) from CpA/K124A/K124Q mutants reveal no noticeable differences in CpC/HJURP levels. **B**) Levels of the mutants are tracked across 8 days, revealing 2d post-transfection accrued the peak level of mutant proteins, allowing us to ChIP at 2 days against the HA-tag to observe any CENP-C binding defects. **C**) Percent distribution of CENP-C/HA-tagged mutant for the fiber IF experiments. **D**) Medium-sized arrays from 4-min MNase-digested chromatin were used in our ChIP assays, and overall comparison between ChIP’ed HA-tagged H3 versus CpA mutant chromatin. kD mark = 20kD, I = Input, U = Unbound. **E**) CENP-C interacts with endogenous CENP-A after ACA ChIP.

**Additional file 7: Fig. S5.** K124A/Q mutants have normal cell ploidy and progress through the cell cycle with no aberrant defects. **A**) Overlaid FACS analysis of GFP-tagged CpA/K124A/Q mutants. **B**) Separated FACS profiles for each mutant and time point. X-axis is the propidium iodide level and the Y-axis is the cell count.

**Additional file 8: Fig. S6.** Identifying CENP-A on double long TAU (DLτ) and long TAU (Lτ) gels for mass spectrometry and HAT/HDAC inhibitor drug treatments. **A**) dLT gels remove excess canonical histone components, leaving behind predominantly CENP-A and histone H2A. Numbers on the DLτ represent bands that were sent for mass spec, and duplicate gel was used for Western and probed against CENP-A. **B**) Whole cell extracts from cells that were untreated, treated with a HAT inhibitor or HDAC inhibitor, were probed against CpA K124ac, CpA, H2A, H4K5ac and H4. The probes were also used against recombinant CpA/H4 (rCpA + rH4) or recombinant chemically ligated K124ac (rK124ac) to determine antibody specificity. **C**) Untreated cells with chromatin-bound, hydroxyapatite-purified histones were ran on a long TAU (LT) gel, and duplicate gel transferred to a membrane was probed for K124ac, H2A and H4.

**Additional file 9: Fig. S7.** A model depicting how cyclical switching in CENP-A K124 acetylation and methylation might affect centromeric replication dynamics. **A**) Model of CENP-A K124me-containing nucleosome. **B**) Cell cycle progression of K124ac during G1/S to K124me at S phase. **C**) Current model encompassing the dynamics of how K124 modifications affect replication and kinetochore protein CENP-C binding, before and during replication.

## Abbreviations

ChIP: chromatin immunoprecipitation
COM: center of mass
HAT: histone acetyltransferase
HDAC: histone deacetylase
IF: immunofluorescence
MD: molecular dynamics
NCP: nucleosome core particle
PCA: principle component analysis
Pre-RC: pre-replication complex
RMSF: root-mean-square fluctuation
TAU/L-TAU (LT)/DLT: triton acid urea/long triton acid urea/double long Triton-Acid-Urea
WB: Western blot
