# Supplementary figures and images for: Internal modifications in the CENP-A nucleosome modulate centromeric dynamics
Source: Epigenetics Chromatin. 2017 Apr 4;10:17. doi: 10.1186/s13072-017-0124-6 (PMC5379712; doi:10.1186/s13072-017-0124-6)

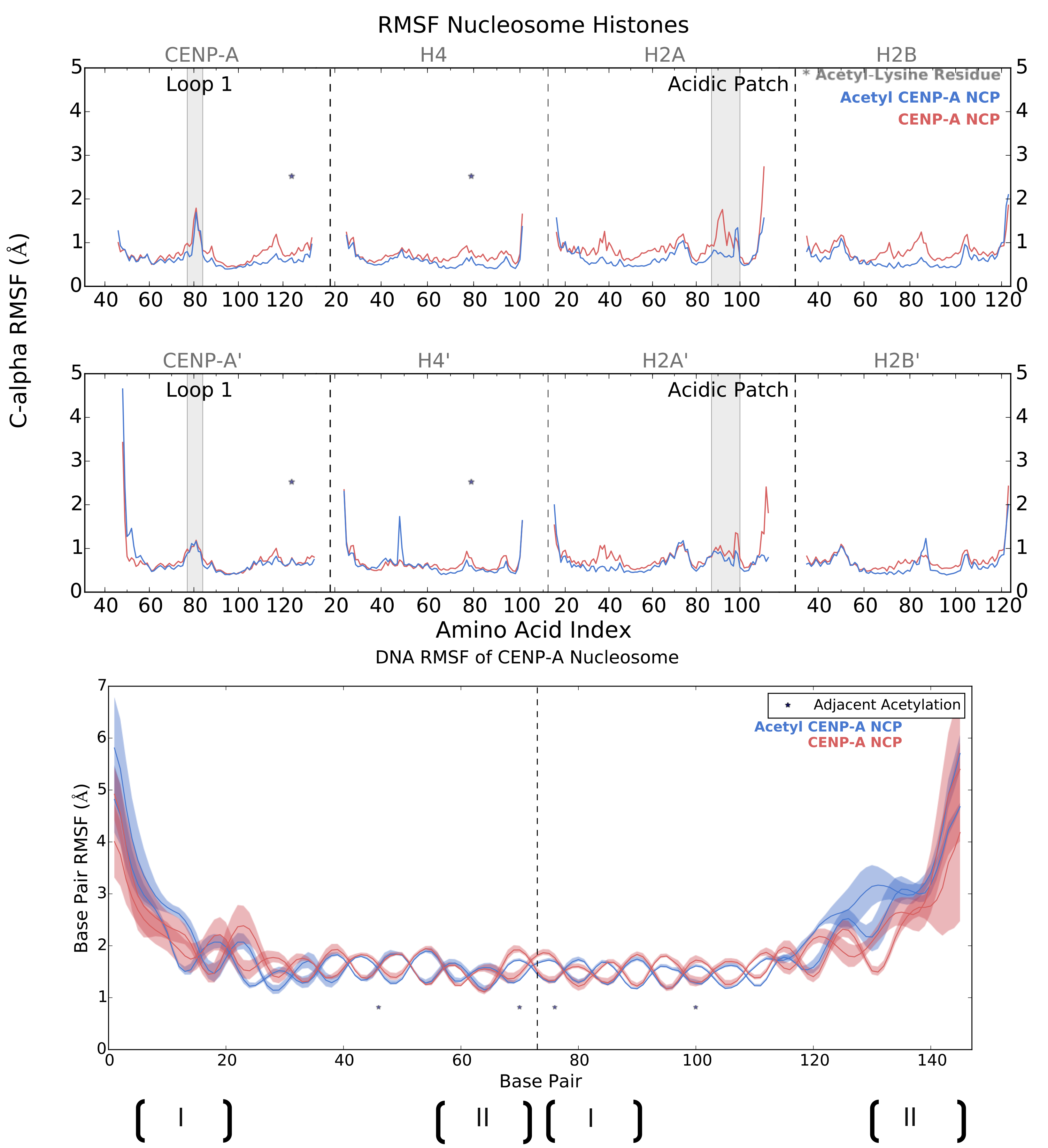

Supplement: Supplementary file 2 — Additional file 2: Fig. S1. RMSF of proteins. A) This decrease in RMSF of Cα residues upon acetylation is more pronounced on the histone heterotetramer adjacent to the entry DNA. Of particular interest, the RMSF of the acetylated H2A acidic patch was suppressed with acetylation by −1 Å, and suppression is shown in the CENP-A C-terminus. The greater similarity shown in the RMSF of the reciprocal histones—CENP-A′, H4′. H2A′, and H2B′—could potentially be explained by the observed asymmetric unwrapping of DNA where the exit end in both systems dissociates to a similar amount (Fig. 3A). B) The RMSF of whole base pairs is shown for each DNA strand. Regions marked by I are DNA wrapped near the entry or near CENP-A and II are near the exit end of CENP-A′. The pseudo-dyad is marked by the vertical dotted line. [file 13072_2017_124_MOESM2_ESM.tif]

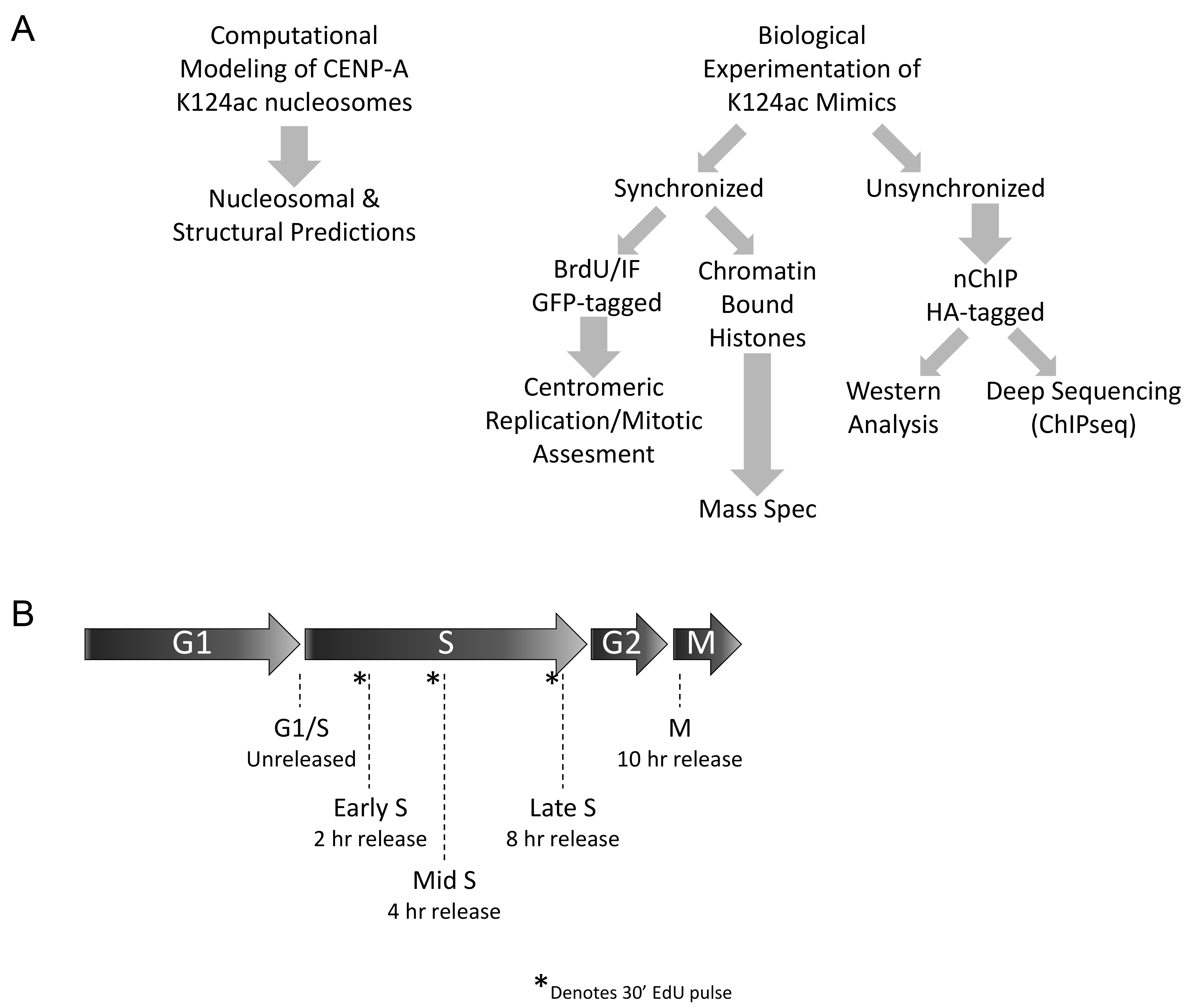

Supplement: Supplementary file 4 — Additional file 4: Fig. S2. Experimental scheme for experiments and cell synchronization. A) Computation and biological experimental scheme for this publication. B) Cell cycle synchronization with a 30-min EdU pulse prior to preparing slides for EdU and immunofluorescence. [file 13072_2017_124_MOESM4_ESM.tif]

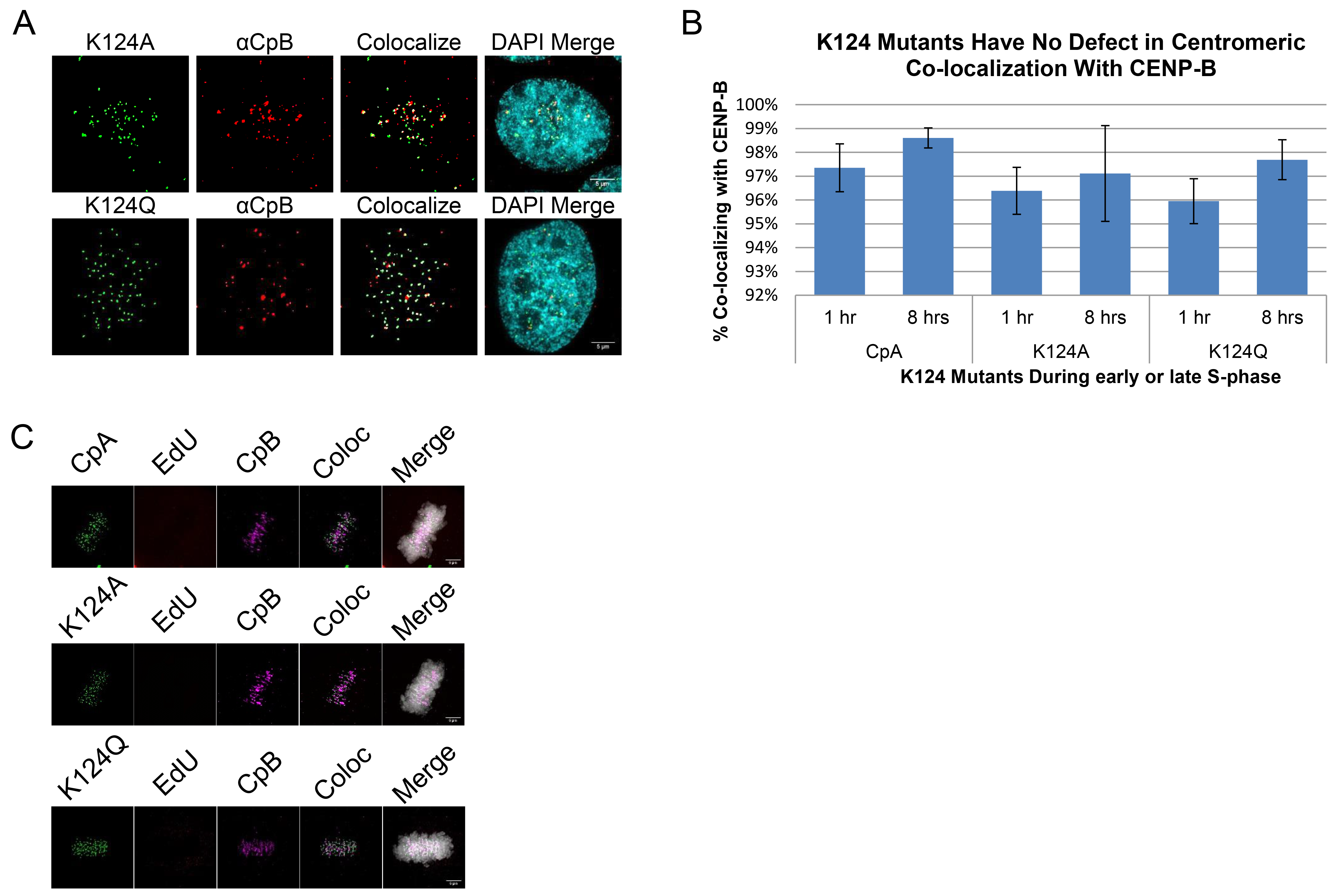

Supplement: Supplementary file 5 — Additional file 5: Fig. S3. Co-CENP-B/CENP-C staining with HA-tagged K124A/Q proteins. A) Unsynchronized cells stained with CENP-B (CpB) and B) percentage of CENP-B co-localizing with GFP-CENP-A (CpA) or K124A/Q exogenously expressed proteins after a double thymidine block and released for 1 or 8 h. C) Co-localization of the GFP-tagged CpA/K124A/K124Q with CpB during metaphase. [file 13072_2017_124_MOESM5_ESM.tif]

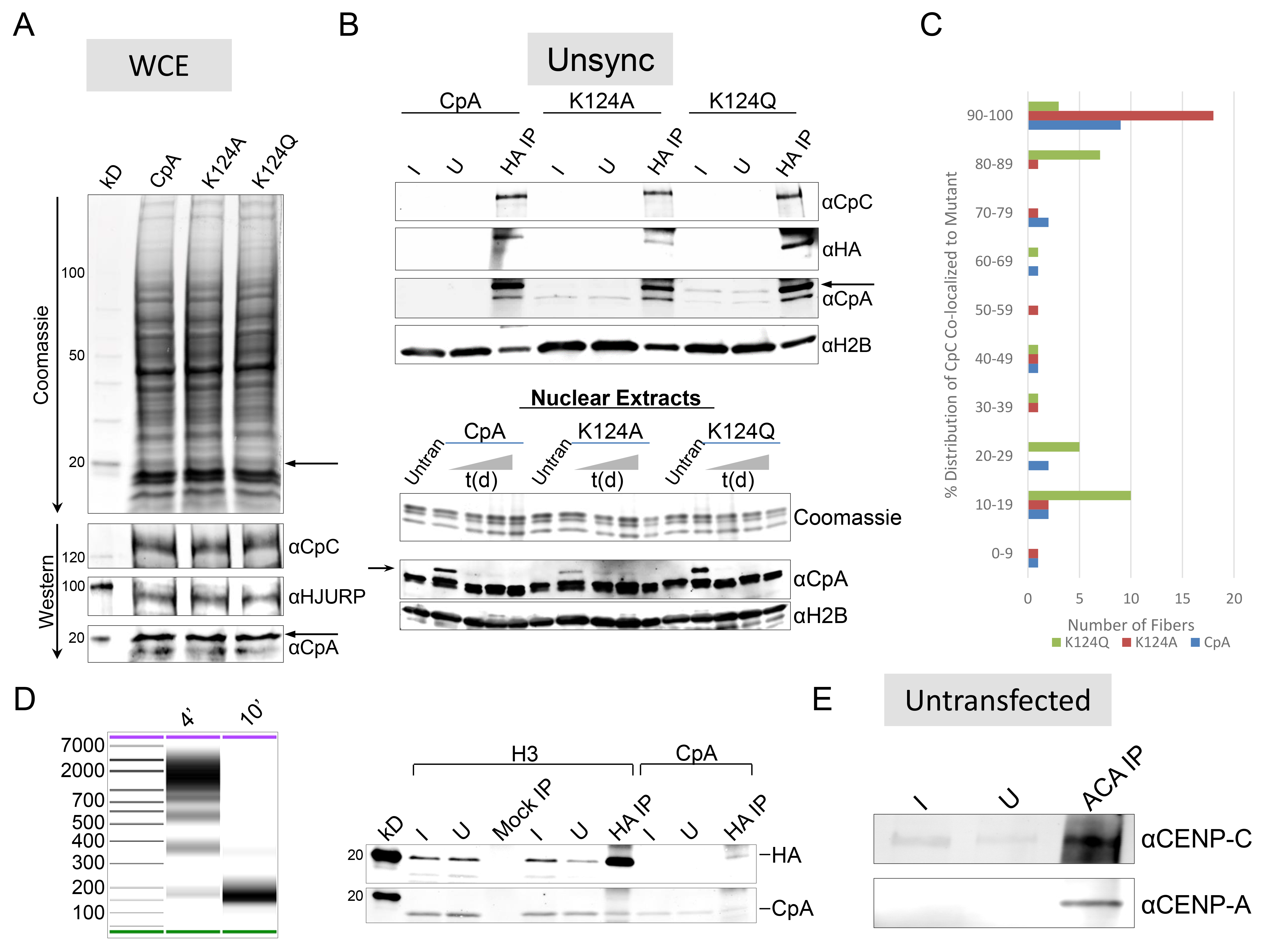

Supplement: Supplementary file 6 — Additional file 6: Fig. S4. K124A/Q have altered affinity for CENP-C. A) Whole cell extracts (WCE) from CpA/K124A/K124Q mutants reveal no noticeable differences in CpC/HJURP levels. B) Levels of the mutants are tracked across 8 days, revealing 2d post-transfection accrued the peak level of mutant proteins, allowing us to ChIP at 2 days against the HA-tag to observe any CENP-C binding defects. C) Percent distribution of CENP-C/HA-tagged mutant for the fiber IF experiments. D) Medium-sized arrays from 4-min MNase-digested chromatin were used in our ChIP assays, and overall comparison between ChIP’ed HA-tagged H3 versus CpA mutant chromatin. kD mark = 20kD, I = Input, U = Unbound. E) CENP-C interacts with endogenous CENP-A after ACA ChIP. [file 13072_2017_124_MOESM6_ESM.tif]

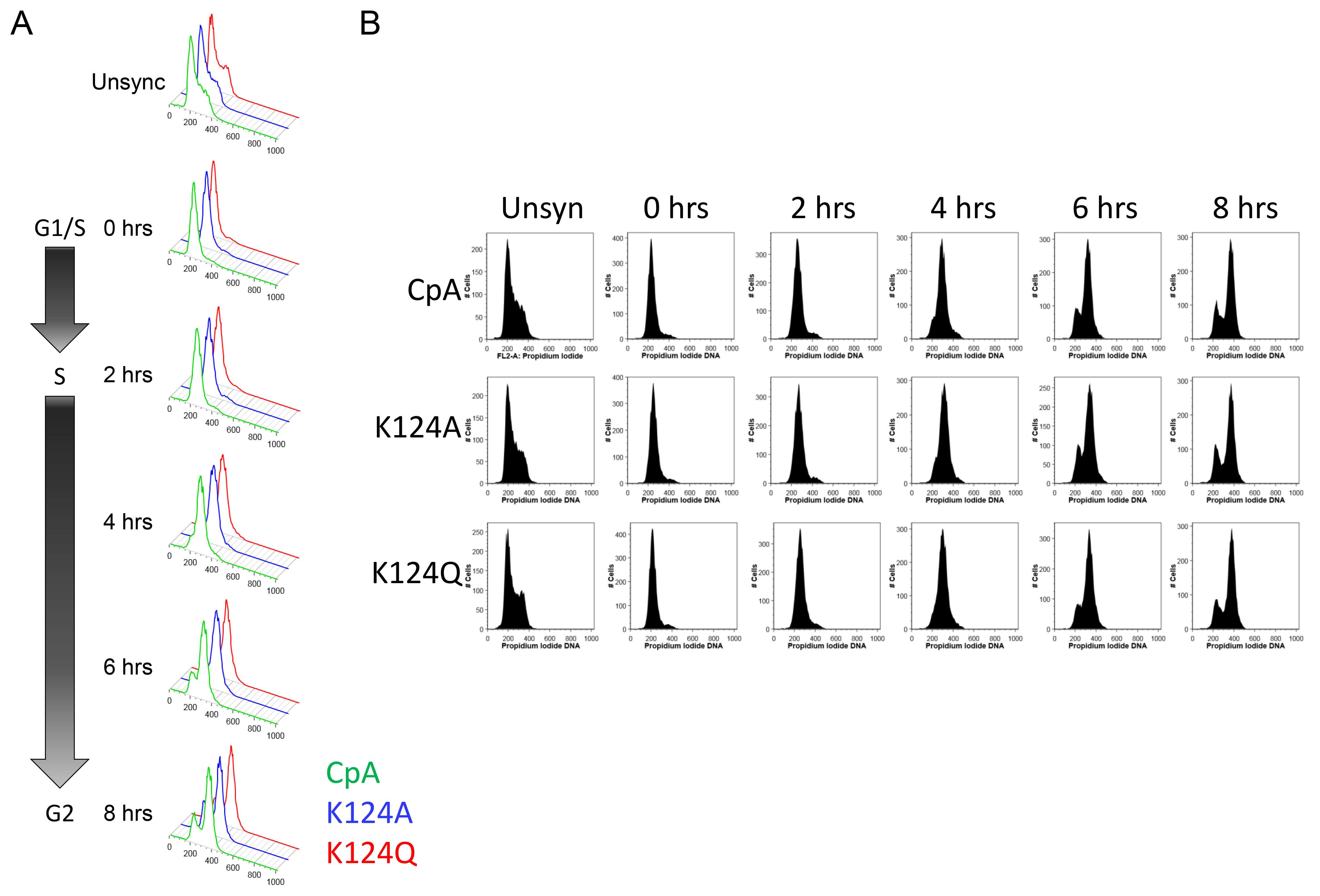

Supplement: Supplementary file 7 — Additional file 7: Fig. S5. K124A/Q mutants have normal cell ploidy and progress through the cell cycle with no aberrant defects. A) Overlaid FACS analysis of GFP-tagged CpA/K124A/Q mutants. B) Separated FACS profiles for each mutant and time point. X-axis is the propidium iodide level and the Y-axis is the cell count. [file 13072_2017_124_MOESM7_ESM.tif]

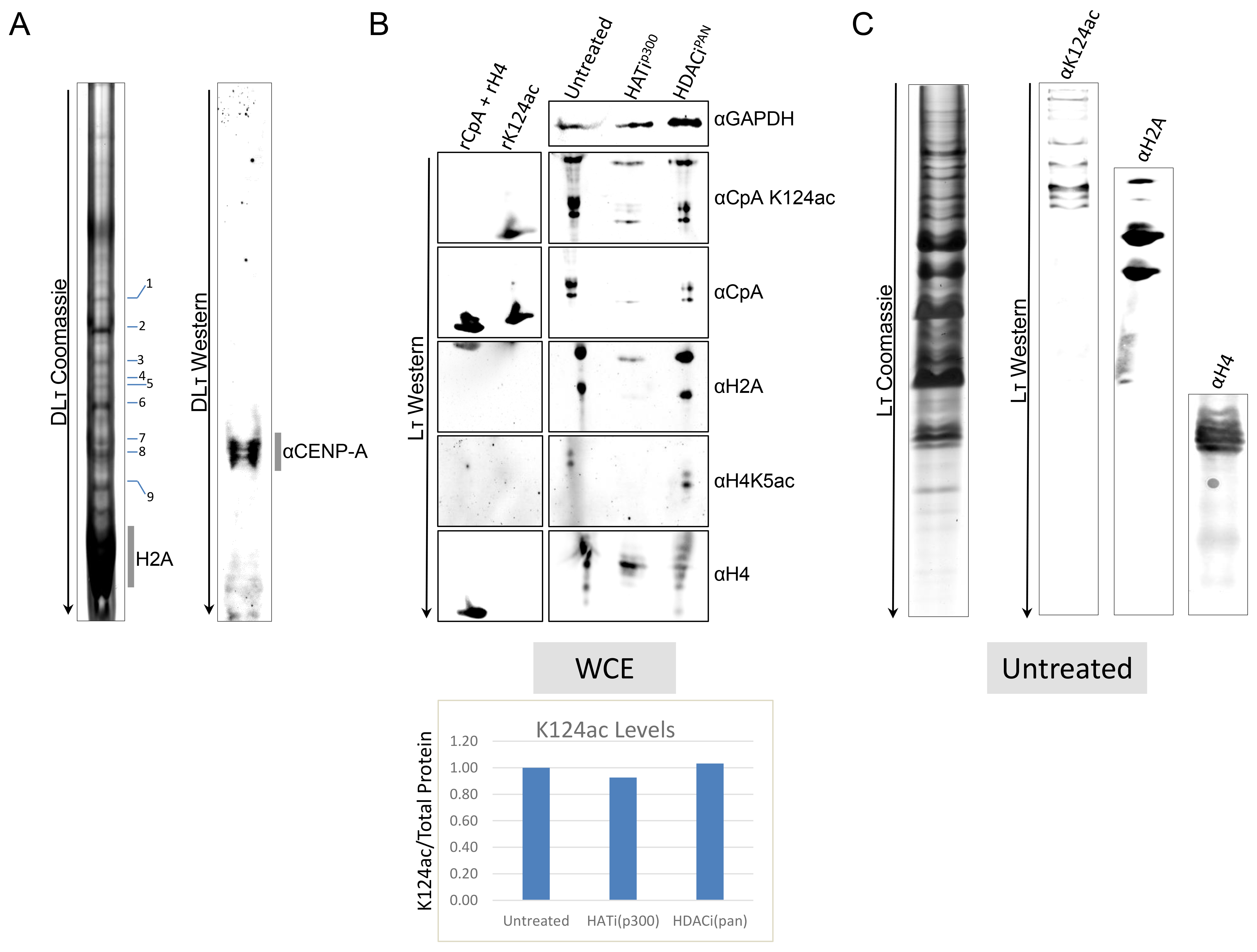

Supplement: Supplementary file 8 — Additional file 8: Fig. S6. Identifying CENP-A on double long TAU (DLτ) and long TAU (Lτ) gels for mass spectrometry and HAT/HDAC inhibitor drug treatments. A) dLT gels remove excess canonical histone components, leaving behind predominantly CENP-A and histone H2A. Numbers on the DLτ represent bands that were sent for mass spec, and duplicate gel was used for Western and probed against CENP-A. B) Whole cell extracts from cells that were untreated, treated with a HAT inhibitor or HDAC inhibitor, were probed against CpA K124ac, CpA, H2A, H4K5ac and H4. The probes were also used against recombinant CpA/H4 (rCpA + rH4) or recombinant chemically ligated K124ac (rK124ac) to determine antibody specificity. C) Untreated cells with chromatin-bound, hydroxyapatite-purified histones were ran on a long TAU (LT) gel, and duplicate gel transferred to a membrane was probed for K124ac, H2A and H4. [file 13072_2017_124_MOESM8_ESM.tif]

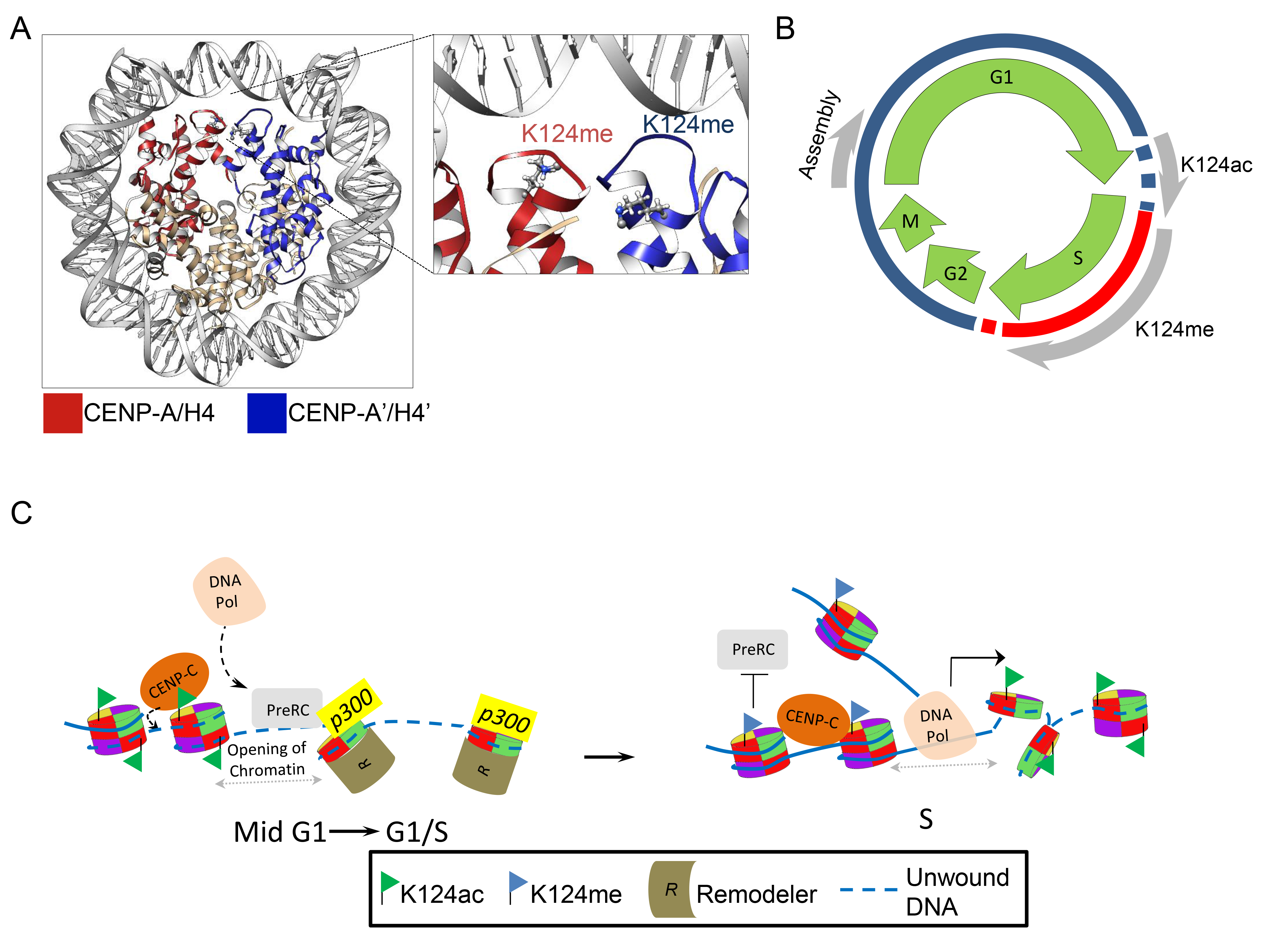

Supplement: Supplementary file 9 — Additional file 9: Fig. S7. A model depicting how cyclical switching in CENP-A K124 acetylation and methylation might affect centromeric replication dynamics. A) Model of CENP-A K124me-containing nucleosome. B) Cell cycle progression of K124ac during G1/S to K124me at S phase. C) Current model encompassing the dynamics of how K124 modifications affect replication and kinetochore protein CENP-C binding, before and during replication. [file 13072_2017_124_MOESM9_ESM.tif]
